# Gene and Genome Parameters of Mammalian Liver Circadian Genes (LCGs)

**DOI:** 10.1371/journal.pone.0046961

**Published:** 2012-10-10

**Authors:** Gang Wu, Jiang Zhu, Fuhong He, Weiwei Wang, Songnian Hu, Jun Yu

**Affiliations:** 1 CAS Key Laboratory of Genome Sciences and Information, Beijing Institute of Genomics, Chinese Academy of Sciences, Beijing, China; 2 Graduate University of Chinese Academy of Sciences, Beijing, China; 3 Department of Pathology, Massachusetts General Hospital and Harvard Medical School, Boston, Massachusetts, United States of America; 4 Laboratory of Disease Genomics and Individualized Medicine, Beijing Institute of Genomics, Chinese Academy of Sciences, Beijing, China; 5 Department of Medicine, University of Alberta, Edmonton, Alberta, Canada; Karlsruhe Institute of Technology, Germany

## Abstract

The mammalian circadian system controls various physiology processes and behavior responses by regulating thousands of circadian genes with rhythmic expressions. In this study, we redefined circadian-regulated genes based on published results in the mouse liver and compared them with other gene groups defined relative to circadian regulations, especially the non-circadian-regulated genes expressed in liver at multiple molecular levels from gene position to protein expression based on integrative analyses of different datasets from the literature. Based on the intra-tissue analysis, the liver circadian genes or LCGs show unique features when compared to other gene groups. First, LCGs in general have less neighboring genes and larger in both genomic and 3′-UTR lengths but shorter in CDS (coding sequence) lengths. Second, LCGs have higher mRNA and protein abundance, higher temporal expression variations, and shorter mRNA half-life. Third, more than 60% of LCGs form major co-expression clusters centered in four temporal windows: dawn, day, dusk, and night. In addition, larger and smaller LCGs are found mainly expressed in the day and night temporal windows, respectively, and we believe that LCGs are well-partitioned into the gene expression regulatory network that takes advantage of gene size, expression constraint, and chromosomal architecture. Based on inter-tissue analysis, more than half of LCGs are ubiquitously expressed in multiple tissues but only show rhythmical expression in one or limited number of tissues. LCGs show at least three-fold lower expression variations across the temporal windows than those among different tissues, and this observation suggests that temporal expression variations regulated by the circadian system is relatively subtle as compared with the tissue expression variations formed during development. Taken together, we suggest that the circadian system selects gene parameters in a cost effective way to improve tissue-specific functions by adapting temporal variations from the environment over evolutionary time scales.

## Introduction

Circadian rhythm controls biological processes in a 24-hour cycle and presents in most organisms from photosynthetic prokaryotes to complex eukaryotes. It is regulated intrinsically in a self-sustainable way and entrained by temporal cues from the environment [1]–[3]. The circadian system offers adaptive advantages to organisms in coping with environmental changes and synchronizing its physiology states to the solar day. A typical circadian system contains hierarchical, multilayered regulatory networks that involve the input system, biochemical and cellular oscillators, and the output system [4]. In mammals, circadian oscillators include the master pacemaker located in the suprachiasmatic nuclei (SCN) [5] and peripheral oscillators present in other organs such as the liver, the heart, and the adrenal glands [6]. Master oscillators in SCN receive photic information from the retina and then transmit rhythmic information to cells in other brain regions and peripheral oscillators through neuronal connections, endocrine signals, and indirect cues initiated from oscillating behavior, and finally coordinated with the peripheral oscillators to drive oscillations in physiology and behavior such as body temperature, hormone secretion, and feeding behavior adaptive to environmental rhythmic variations [7], [8]. Cell-autonomous oscillations in both central and peripheral organs are mainly generated by the core circadian network comprised of interlocked transcriptional-translational feedback loops and their directly/indirectly regulated genes [9], [10], and such a network may be influenced even by small molecules [11].

Since 2002, a series of microarray-based transcriptomic studies have been conducted for genome-wide identification of circadian oscillating genes from different tissues of mammalian species, especially from murine tissues [12], [13]. Numerous circadian genes have been identified and defined in the same or different species although discrepancies about the number of circadian genes from different experiments do exist due to differences in experimental designs and computational tools used [14]. Efforts have been made to improve the ability of identifying circadian genes more precisely by using different approaches, such as combining different experiments but based on the same analysis protocol [15], using novel experimental design for high-density temporal sampling [16], and developing novel algorithms for better data analysis [3], [17]. Along with these improvements, there are two obvious and yet consistent results. First, there have been more circadian genes identified than previously anticipated, and ∼10,000 circadian genes have been meta-recognized in mice [15], and over 3,000 circadian transcripts are precisely identified in the murine liver [18]. Second, there are non-rhythmically expressed genes—not defined as circadian genes based on the current methods—that are now tentatively named as the non-circadian gene or NCGs. The NCG group provides an optimal control set for studying features of circadian genes and associated regulatory mechanisms. However, we have to be cautious in classifying circadian and NCGs as other than expression patterns there have been a limited number of distinctions in genome-scale parameters between the two gene groups. Our hope here is to ascertain useful clues and regulatory details of the circadian system through comparative analysis on various genome parameters distinctive primarily between the two groups, often based on their statistic significances.

Data from high-density temporal sampling of murine liver, pituitary glands, and NIH3T3 cells [16], [18], [19] provide essential materials for precise and recurring identification of circadian genes based on novel algorithms [17], [20]. For comparative analysis, there are also other experimental datasets, especially those suitable for meta-analysis (such as transcriptomic and proteomic studies on multiple murine tissues) [21], [22]. Furthermore, data from the liver, the most important mammalian peripheral circadian organ, has been serving as primary information since the liver gene expression is largely driven by circadian clock and temporal pattern of food intake [19], [23]. Liver circadian oscillators help an organism adapting a daily pattern tailored to food intakes through circadian-tuned expression of genes involved in regulating metabolic and physiological activities. In fact, mammals that lack functional liver circadian clock under experimental conditions often encounter various metabolic dysfunctional diseases [24], [25]. Therefore, studying the liver circadian genes (LCGs) at multiple levels is of essence in understanding how peripheral circadian oscillators regulate metabolism and physiology in the liver and other vital organs/tissues.

In this study, we first identified all circadian-regulated transcripts based on a microarray dataset from high-density temporal sampling of the murine liver, using JTK_CYCLE [17] and HAYSTACK [20]. We went on to re-define LCGs and two other datasets—non-liver circadian genes (NLCGs) and liver-expressed non-circadian genes (LNCGs)—based on our new analysis strategies. We also validated specificities of LCGs and LNCGs based on the literature. Our results show that LCGs exhibit special characteristics when compared to liver-expressed NLCGs and LNCGs, especially in genomic parameters and expression features, and all offer information on the superiority of circadian genes in performing highly orchestrated tissue-specific functions.

## Results

### The re-definition of circadian and non-circadian gene sets based on public data from murine liver

**Figure 1:**
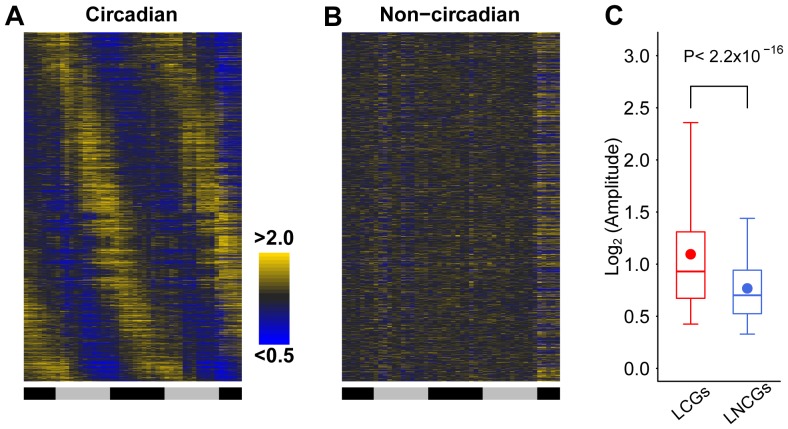
Expression profiles and amplitudes of the murine LCGs and LNCGs. A heatmap shows temporal expression profiles of LCGs (A) and LNCGs (B) based on median-normalized values. Expression levels are segregated into at least 2-fold or more (yellow) and 50 percent or less (blue) than the median intensity values, respectively. The horizontal bars indicate temporal phases in circadian day (black) and night (grey). The amplitude (C) of LCGs (red) or LNCGs (blue) are shown in a box plot, which is estimated by calculating the peak-to-trough ratio ( = percentile[0.95, x]/percentile[0.05, x]). The boxes depict data between the 25th and 75th percentiles with central horizontal lines and solid circles representing the median and mean values, respectively, and with whiskers showing the 5th and 95th percentiles. *P*-values are calculated based on the Wilcoxon rank sum test.

We re-analyzed microarray data from the murine liver using JTK_CYCLE and HAYSTACK, selected the transcripts using a q-value threshold of <0.001, and mapped them to the mouse genome RefSeq loci. The protocol yielded 1,888 circadian genes (Figure 1A and Table S1) with a false positive rate of 0.9%, bench-marked based on 111 negative control genes (Table S2). We selected 1,701 non-circadian genes expressed in the liver, i.e., liver-expressed non-circadian genes or LNCGs (Figure 1B and Table S1), with a false positive rate of 1.9%, estimated based on 104 literature-supported circadian genes (Table S2). The mean amplitude of LCGs is 2.6, while 79.4% of LNCGs with a mean amplitude less than 2 (Figure 1C). All selected genes have maximal expression values (using logarithm of intensity to base 10) above 1.45 based on the density plot (Figure S1A) and are validated to be practical by comparing with RNA-seq data (Figure S1B).

We also performed gene ontology (GO) analysis on both LCGs and LNCGs. LCGs appear specifically enriched in biological process related to protein polymerization, cellular carbohydrate biosynthetic process, response to hormone stimulus, steroid metabolic process, protein folding and nitrogen compound biosynthetic process, selectively located in peroxisome, and associated with the molecular function of unfolded protein binding (Figure S2A; *P*<0.01 and enriched fold >2). LNCGs seem specifically enriched in the biological process related to tRNA metabolic process and associated with the molecular function of tRNA binding and N-methyltransferase activity (Figure S2B; *P*<0.01 and enriched fold >2).

### The chromosomal distribution of LCGs shows relative isolation from clustered genes

**Figure 2:**
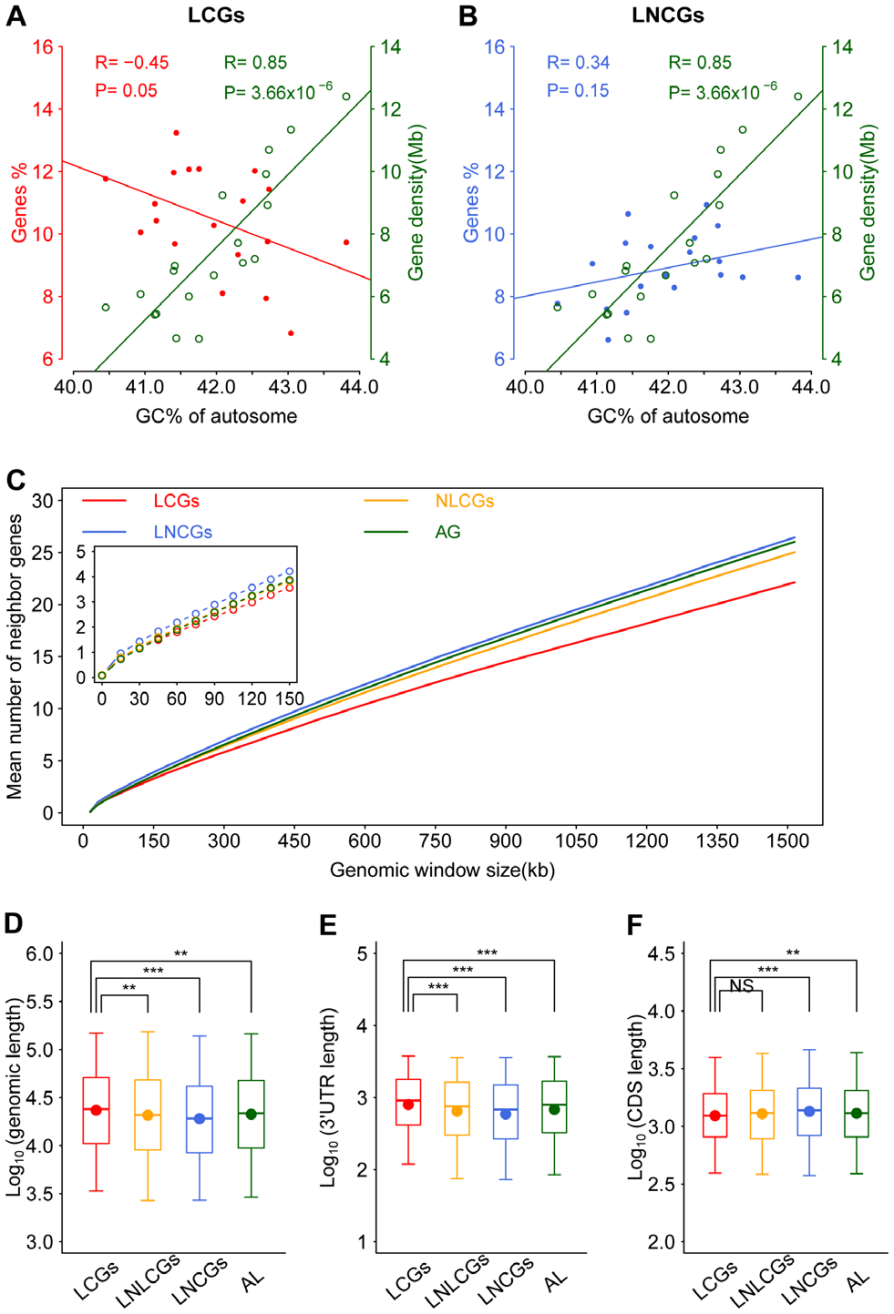
Chromosome distribution and gene parameters of the murine LCGs. The percentage of LCGs (A, red solid circles) or LNCGs (B, blue solid circles), and gene density (open green circle) against the GC content of each autosome are plotted together. The gene density is defined as the mean gene numbers per megabase (Mb) in a chromosome. The average numbers of neighboring genes (C) for LCGs (red), non-liver circadian genes (NLCGs, orange), LNCGs (blue) or all genes in the genome (AG, green) are calculated in a given genomic length window (from zero to 1.5 Mb with a step length of 15 kb). The inset shows the portion from zero to 150 kb. Other genomic parameters include genomic length (D), 3′-UTR length (E), and CDS length (F) for LCGs, liver-expressed NLCGs (LNLCGs), LNCGs, and all liver-expressed genes (AL). The boxes depict data between the 25th and 75th percentiles with central horizontal lines and solid circles representing the median and mean values, respectively, and with whiskers showing the 5th and 95th percentiles. *P*-values are calculated based on the Wilcoxon rank sum test. (**), *P*-value<0.01. (***), *P*-value<0.001. NS, not significant.

Gene density is a genome parameter, which positively correlates with chromosomal GC content (Figure 2A and B; R = 0.85, *P* = 3. 66e-6) but LCGs appear taking an opposite trend—negatively correlate with GC content (Figure 2A; R = −0.45, *P* = 0.05). The enrichment of LCGs in AT-rich chromosomal regions suggests that they are not clustered in GC-rich regions but scattered over GC-poor or AT-rich regions. In contrast, the correlation coefficient of LNCGs vs. chromosomal GC content (Figure 2B; R = 0.34, *P* = 0.15) is positive albeit insignificant in a statistics sense.

We further compared the mean number of neighboring genes among the four datasets, i.e., LCGs, NLCGs, LNCGs, and all genes, with variable window sizes from zero to 1.5 Mb in a step length of 15 kb. LCGs show less neighboring genes than all three other groups on average (Figure 2C), i.e., the mean number of neighboring genes for LCGs, NLCGs, LNCGs, and all genes are 3.6, 3.8, 4.2 and 3.9 neighbors in the window size of 150 kb, respectively; even in a smaller, such as a 60-kb window, the numbers are 1.8, 1.9, 2.2, and 1.9 for the four groups, respectively (inset of Figure 2C). Our results suggest that LCGs, and circadian genes in general, are not as densely packed throughout the genome as other groups of genes are, especially distinguishable from LNCGs. The less densely packed feature might be the superiority of circadian genes for harboring more regulatory elements. We therefore further studied the regions around the transcription start site (TSS) of LCGs and LNCGs. We found that LCGs contain more E-boxes (Figure S3A) and strong CpG islands (Figure S4A) than LNCGs in the promoter regions. We also noticed that genes containing E-box have less neighboring genes on average than those without E-box regardless if they are LCGs or LNCGs, and that LCGs always contain less neighboring genes with or without E-box (Figure S3B). Interestingly, DNA methylation levels of LCGs are always lower than those of LNCGs in their promoters, and show significant different in weak CpG islands and CpG poor classes (Figure S4B; *P*<0.01).

Although LCGs are in general scattered over the chromosomes, there are still a limited number of special loci where circadian genes are potentially forming clusters in the chromosomes. We identified 19 divergently-paired circadian genes with phase difference no more than 6 hours in the liver (Table S3). Interestingly, three of the divergently-paired circadian genes (*Hnrpa2b1*/*Cbx3*, *Tmem93*/*Tax1bp3*, and *Pigf*/*Cript*) are shared by the adrenal glands.

### LCGs are relative larger but encode smaller proteins in general

In terms of gene structure, LCGs are significantly longer in genomic (Figure 2D; *P*<0.01) and 3′-UTR lengths (Figure 2E; *P*<0.001), but shorter in CDS length (Figure 2F; *P*<0.01) as compared to LNCGs and all liver-expressed genes. Comparing with liver-expressed NLCGs, LCGs are also longer in genomic (Figure 2D; *P*<0.01) and 3′-UTR lengths (Figure 2E; *P*<0.001), and shorter in CDS length but not significant (Figure 2F). The medians of genomic, 3′-UTR, CDS lengths are 24.0 kb, 0.91 kb, and 1.2 kb for LCGs, 20.9 kb, 0.76 kb, and 1.3 kb for liver-expressed NLCGs, 19.1 kb, 0.68 kb, and 1.4 kb for LNCGs and 21.7 kb, 0.80 kb, and 1.3 kb for all liver-expressed genes, respectively (Figure 2D–F). The shorter CDS length of circadian genes indicates that the circadian system prefers to regulate genes encoding small proteins, for which the energy cost is relatively lower. Alternatively, the circadian genes may be evolutionarily selected to have such features for some other reasons.

Comparing with LNCGs, we questioned if longer 3′-UTR of LCGs may contain more regulation elements, such as microRNA targets. We compared the number of predicted microRNA targets between the two groups, and found that LCGs have significant more predicted microRNA targets in their 3′-UTR sequences than what in LNCGs (Figure S5A; *P*<0.01), with medians of 16 and 14, respectively, which indicated that circadian genes may be more frequently regulated by microRNA than non-circadian genes at least in the liver. As to 5′-UTRs, LCGs have significantly longer length than that of LNCGs (*P*<0.001), but we did not find significant more regulation elements such as upstream open reading frames or uORF in the 5′-UTRs of LCGs (data not shown). The result indicates that the 3′-UTR length may be more important than the 5′-UTR length in circadian regulation as 3′-UTRs may harbor more regulatory elements and thus provide adequate rooms for more sophisticated regulation.

### LCGs are in general highly expressed with higher degree of expression variations

**Figure 3:**
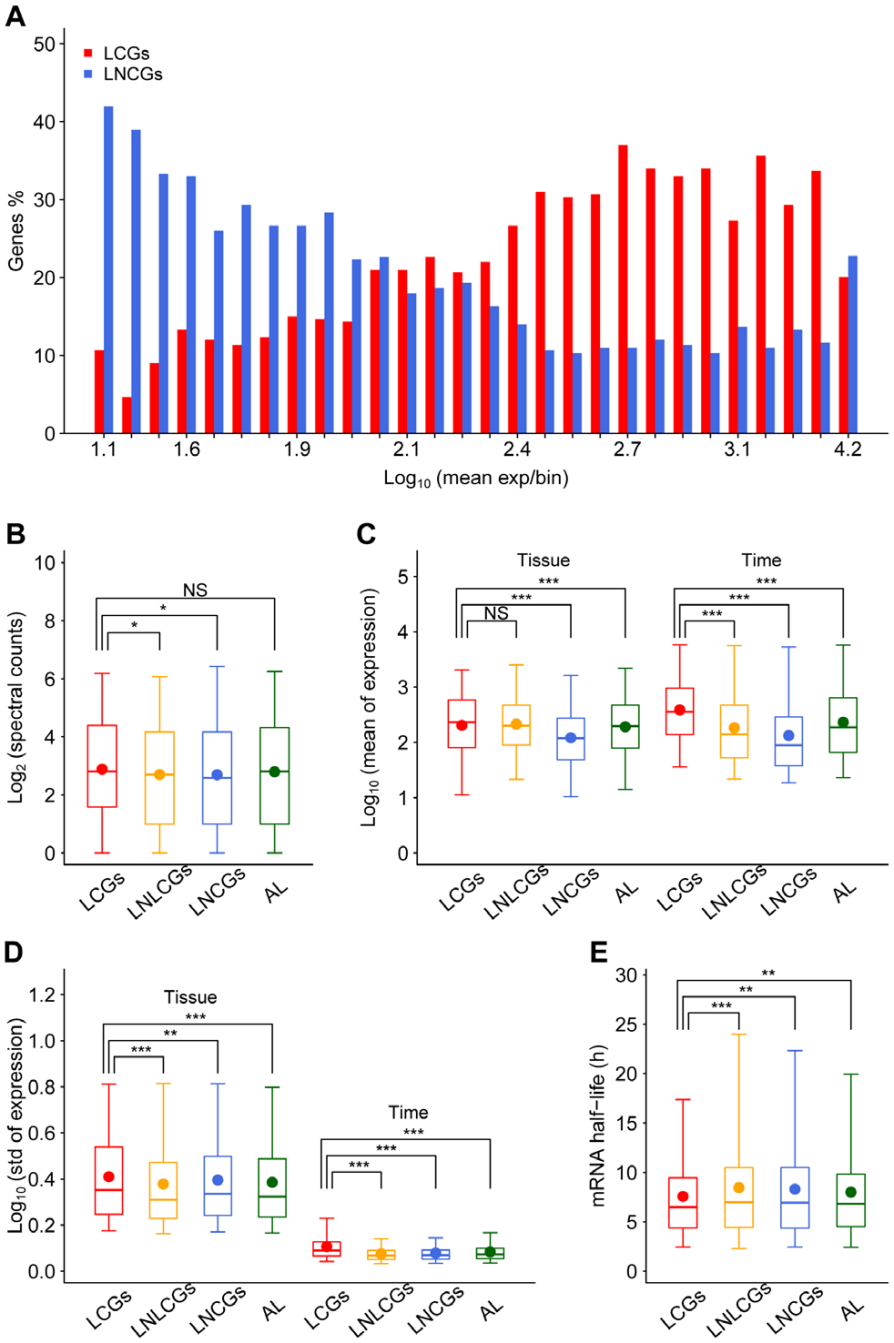
Expression abundance and dynamics of murine LCGs. (A) LCGs (red) and LNCGs (blue) in each expression category. All liver-expressed genes are ranked according to their expression levels (by using logarithm of intensity to base 10) and divided into 28 expression bins with each bin containing 300 genes except the highest one with 329 genes. The x-axis showed the mean expression value of each bin. (B) Abundance (using logarithm of spectral counts to base 2) of proteins encoded by LCGs, liver-expressed non-liver circadian genes (LNLCGs), LNCGs, and all liver-expressed genes (AL). (C) Mean expression values of LCGs, LNLCGs, LNCGs, and all liver-expressed genes among different tissues or temporal phases in the liver. (D) Standard deviations of LCGs, LNLCGs, LNCGs, and all liver-expressed genes among different tissues or temporal phases in the liver. (E) mRNA half-life of LCGs, LNLCGs, LNCGs, and all liver-expressed genes in ES cells. The boxes depict data between the 25th and 75th percentiles with central horizontal lines and solid circles representing the median and mean values, respectively, and with whiskers showing the 5th and 95th percentiles. *P*-values are calculated based on the Wilcoxon rank sum test. (*), *P*-value<0.05. (**), *P*-value<0.01. (***), *P*-value<0.001. NS, not significant.

LCGs are not only concentrated in expression bins with higher mRNA abundance (Figure 3A) but also show significantly higher protein abundance (Figure 3B; *P* = 0.02) than LNCGs. Among the expression bins with average expression levels higher than 2.1 (other than in the highest one), there are more LCGs than LNCGs (Figure 3A), and the former reach the highest percentage (37.0%) in a bin at the expression level of 2.7. These results suggest that the circadian system prefers to regulate genes with moderate-to-high expression levels.

We further compared expression features of LCGs with liver-expressed NLCGs, LNCGs, and all liver-expressed genes by incorporating transcriptomic data from 46 different tissues. The mean expression values of LCGs (2.31 among tissues and 2.59 across time points in the liver) are significantly higher than those of LNCGs (2.09 among tissues and 2.13 across time points in the liver; Figure 3C; *P*<0.001) and all liver-expressed genes (2.28 among tissues and 2.37 across time points in the liver; Figure 3C; *P*<0.001). Compared with liver-expressed NLCGs, the mean expression values of LCGs are only significant higher across time points in the liver (Figure 3C; *P*<0.001). The standard deviations (STD) of expression levels are also significantly higher (Figure 3D; *P*<0.01 among tissues and *P*<0.001 across time points) in LCGs (0.41 among tissues and 0.11 across time points in the liver) than liver-expressed NLCGs (0.38 among tissues and 0.08 across time points in the liver), LNCGs (0.40 among tissues and 0.08 across time points in the liver) and all liver-expressed genes (0.39 among tissues and 0.08 across time points in the liver).

The higher expression variation of LCGs across time points suggests more dynamic regulation by the circadian system. Considering that the half-life of mRNAs is closely related to the steady-state concentration of transcripts in cells, we analyzed the half-life of LCG mRNAs in murine ES cells. The mean half-life of the LCGs (7.6 h) is significantly (Figure 3E; *P*<0.01) shorter than that of liver-expressed NLCGs (8.5 h), LNCGs (8.3 h) and all liver-expressed genes (8.0 h). The shorter half-life of LCGs in the undifferentiated cells suggests that mRNA instability of LCGs may be associated with their specific gene parameters that are selected over evolutionary time scales.

### Circadian genes are ubiquitously expressed but only rhythmical in specific tissues

**Figure 4:**
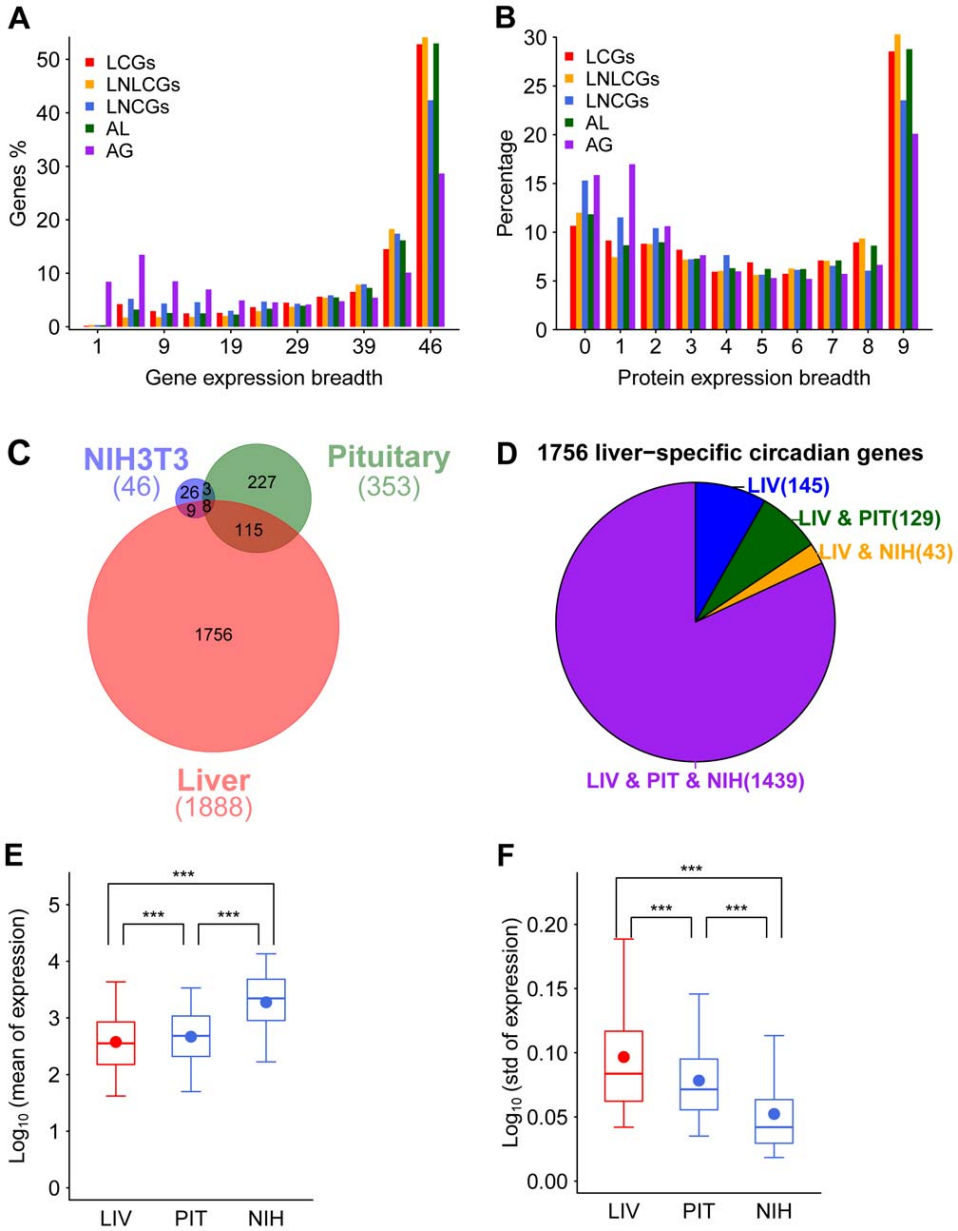
The dualistic features of murine circadian genes. (A) Expression breadth of LCGs (red), liver-expressed non-liver circadian genes (LNLCGs, orange), liver-expressed non-circadian genes (LNCGs, blue), all liver-expressed genes (AL, green), and all genome-wide expressed genes (AG, purple) among 46 different tissues and organs. The breadths from 0 to 46 are divided into 11 bins with five numbers in each bin. The exceptions are: the first bin that has 0 and 1; in the last two bins, one has 4 numbers, from 42 to 45, and the other has the number 46. (B) Expression breadths of proteins encoded by LCGs (red), LNLCGs (orange), LNCGs (blue), all liver-expressed genes (AL, green), and all genome-wide expressed genes (AG, purple) among nine different tissues and organs. (C) Venn diagrams display the relationship of circadian genes among the liver (red), the pituitary gland (green), and NIH3T3 cells (blue) based on data from high temporal resolution profiling. (D) In-depth analysis of 1,756 liver-specific circadian genes expression in four subgroups: genes expressing only in the liver (LIV, blue), in both the liver and the pituitary gland (LIV & PIT, green), in both the liver and NIH3T3 cells (LIV & NIH, orange), and in all three samples (LIV & PIT & NIH, purple). Mean expression values (E) and standard deviations (F) of the three-samples expressed liver-specific circadian genes among different time points in the liver (LIV, red), pituitary gland (PIT, blue), and NIH3T3 cells (NIH, blue). The boxes depict data between the 25th and 75th percentiles with central horizontal lines and solid circles representing the median and mean values, respectively, and with whiskers showing the 5th and 95th percentiles. *P*-values are calculated based on the Wilcoxon rank sum test. (***), *P*-value<0.001.

Similar with liver-expressed NLCGs and all liver-expressed genes, more than half of the LCGs are expressed in all 46 tissues, whereas only 42.3% of LNCGs and 28.6% of all genes in the mouse genome are ubiquitously expressed in all tissues (Figure 4A). In addition to transcriptomic comparisons, we also investigated the expression breadth at the proteomic level, considering that most genes function as proteins and there is partial positive correlation in expression between the protein and transcript levels. 50.3%, 53.0%, 42.3%, 50.7% and 37.7% of proteins encoded by LCGs, liver-expressed NLCGs, LNCGs, all liver-expressed, and all genes, respectively, are detectable in at least six tissues (Figure 4B). However, some LCGs that are ubiquitously expressed in multiple tissues at both transcriptomic and proteomic levels do not mean that they are also rhythmically expressed in other tissues. We compared circadian genes identified from mouse NIH3T3 cells, the pituitary gland, and the liver, and found that only eight circadian genes are shared by all three samples (Figure 4C), and 56.5%, 64.3%, and 93.0% of circadian genes are specific to the three cell/tissue types, respectively. Therefore, the majority of circadian genes tag along rhythmical expression only in a cell-/tissue-specific manner. We studied this dualistic characteristic of LCGs at the transcript level. Of 1,756 liver-specific circadian genes, a great majority of them, 1,439 are also expressed in NIH3T3 cells and the pituitary gland (Figure 4D). The mean expression value in the liver is the lowest in the three samples, but the STD is the highest among the three samples (Figure 4E and F), which indicates that there may be a liver-specific circadian regulation mechanism that restricts the expression level and improves the temporal expression variations of those LCGs with arhythmical expression pattern in NIH3T3 cells and the pituitary gland.

### The temporally co-expressed LCG clusters are highly selected by the circadian system

**Figure 5:**
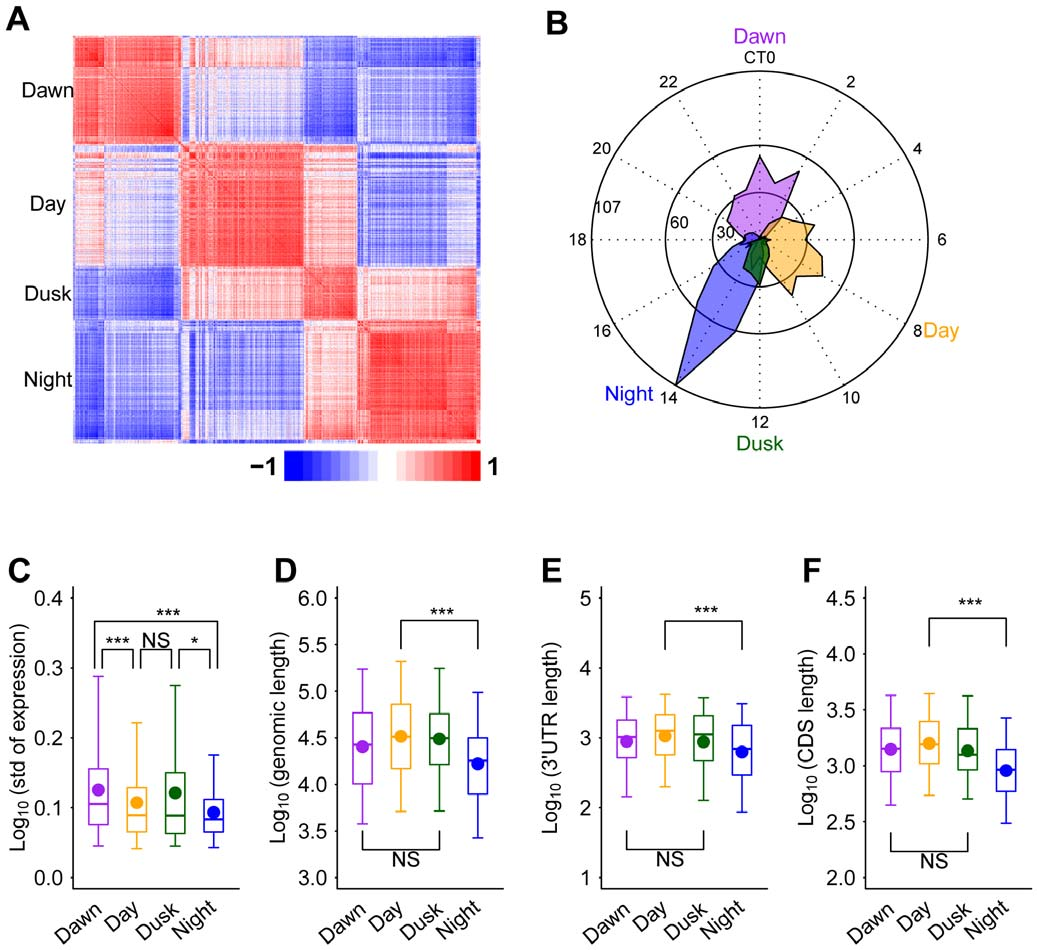
Features and functional analysis of temporal co-expression clusters of LCGs. (A) The heatmap exhibits the four co-expression clusters of the LCGs. Bright red and blue represent co-expression value as 1 and -1, respectively. In each cluster, the gene order ranked based on the consensusNMF result. The four clusters are arranged by their temporal phases and named as the dawn, the day, the dusk, and the night clusters. (B) The number of genes per phase in the four liver clusters. The radial plot displays phases (h) on the circumference and the number of genes on the radius. (C) Standard deviations of the phase-associated genes (dawn, day, dusk, and night) of the liver among different time points are shown in different color-coded boxes (purple, orange, green and blue boxes, respectively). The genomic length (D), 3′-UTR length (E) and CDS length (F) of the dawn, the day, the dusk, and the night clusters are shown in purple, orange, green, and blue boxes, respectively. The boxes depict data between the 25th and 75th percentiles with central horizontal lines and solid circles representing the median and mean values, respectively, and whiskers showing 5th and 95th percentiles. *P*-values are calculated based on the Wilcoxon rank sum test. (*), *P*-value<0.05. (***), *P*-value<0.001. NS, not significant.

As most of mammalian genes are actually regulated as clusters (also known as co-linearity), we further investigated how the circadian system functionally orchestrates the genes and their clusters in a tissue-specific manner. Using the nonnegative matrix factorization (NMF) clustering method, we obtained four major temporal co-expression gene clusters from LCGs as the dawn (phases mainly from CT22 to CT2), the day (phases mainly from CT5 to CT10), the dusk (phases mainly at CT12), and the night (phases mainly from CT13 to CT16) gene clusters (Figure 5A and B). Interestingly, the expression variations in the dawn and dusk clusters (mean STDs 0.13 and 0.12, respectively) are higher than those of the day and night clusters (mean STDs 0.11 and 0.09, respectively; Figure 5C). Higher expression variations in the dawn and dusk clusters may be associated with their close relationships with the light signal transduction. Interestingly, we found that one (mmu−miR−1187; Figure S5B) and four microRNAs (mmu−miR−466d−3p, mmu−miR−148b, mmu−miR−466j, and mmu−miR−411; Figure S5C) are specially enriched in the day and dusk clusters, respectively. This result indicates that microRNA may participate into the phase-specific regulation. In addition, the genomic, 3′-UTR, and CDS lengths of the day cluster are significant longer (*P*<0.001) than those of the night cluster (Figure 5D–F). Our results show that the circadian system appears selecting larger genes (encoding large proteins) to express in an inactive time period (light on) and shorter genes (encoding small proteins) to express in an active time period (light off).

Finally, we carried out gene ontology (GO) analysis on functional annotation of each gene cluster. The significantly enriched functional categories of biological processes are steroid biosynthetic process, sex differentiation, and negative regulation of apoptosis in the dawn cluster, protein catabolic process and response to insulin stimulus in the day cluster, and translation, ribonucleoprotein complex biogenesis, ribosome biogenesis, generation of precursor metabolites and energy, and electron transport chain in the night cluster (Figure S6A–C; at least three out of four pair comparisons with *P*<0.05 and enriched fold >1.5). However, we did not find any specially enriched biological processes in the dusk cluster.

## Discussion

In this study, we sought to study special features of LCGs based on comparison with other groups of genes, especially LNCGs. First, it is reported that gene order in the genome is not random [26] and genes are actually clustered, forming domain structures that have higher gene density, higher GC content, and shorter intron length [27]. LCGs show a less-clustering feature and enjoying residing in chromosomal regions where gene density and GC content are both low, contain larger introns, and have less neighboring genes. This cluster-avoiding behavior may be helpful for containing sufficient regulatory elements (Figure S3A and S4A). Second, it is reported that introns and intergenic regions are regulated by the circadian system in plants [28], and therefore longer genomic length (mainly contributed by longer intron region) and cluster-avoiding positioning (mainly contributed by longer intergenic region) of circadian genes may contain more intra-intronic regulatory elements or non-coding RNA, which may all be beneficial for ensuring fine-tuned temporal tissue-specific regulation of the circadian system. The longer 3′-UTR length is suggested to contribute to mRNA instability in mammals [29], and more microRNA targets are predicted in the 3′-UTR region of LCGs than that of LNCGs (Figure S5A). Thus longer 3′-UTR length in LCGs may be helpful for keeping higher degree of expression variations. In addition, the length variation at 5′-UTR is not significant correlated with gene expression characteristics [30], and it is yet to know if the longer 5′-UTR for LCGs is actually functionally meaningful. Third, shorter CDS, often encoding smaller proteins to facilitate more efficient translation [31], has advantage for the circadian regulation at the protein level, especially when half of the proteins encoded by circadian-regulated transcripts are synthesized and degraded under the influence of the circadian system [32]. After all, we suggest that the unique genomic parameters of circadian genes offer advantages for the circadian system to regulate.

Since stochastic gene expression is omnipresent and highly expressed genes show less expression noise [33], the relative high-level expression of LCGs (Figure 3A and C) may be necessary for a more precise circadian regulation. Furthermore, we also noticed that the fraction of circadian genes is actually lower than that of non-circadian genes in the highest expression bin of the liver genes (Figure 3A) and the mean expression levels of the liver-specific rhythmic genes are significant higher in the pituitary gland and NIH3T3 cell than in the liver (Figure 4E). Therefore, we suggest that circadian genes are selected to have a moderate gene expression level for an effective regulation by the circadian system. However, current methods are not adequate enough for the identification of all circadian genes at low expression levels for the high noise to signal ratios intrinsic to the platform. We expect the expression features of LCGs to be further validated by information from ample temporal RNA-seq data.

Other than expression abundance, expression variation in response to internal or external stimulations is another essential gene expression feature. High temporal expression variations of the core clock genes are necessary for their key roles as molecular oscillator [34], [35]. Aside from tissue specificity, temporal expression variations of LCGs are always higher than those of LNCGs, and we therefore suggest that the high expression variation represents a way where circadian genes are regulated in a noise-minimized context for robust rhythmic physiology or behavior. High expression variations of circadian genes reflect fast accumulation-degradation cycles of their transcripts [36]. Interestingly, we observed that circadian transcripts identified in the liver tend to have shorter half-lives than the LNCGs control in ES cells where there is no established circadian system [37], [38] until differentiated into the liver and adrenal glands [39]. Since protein subunits with longer transcript half-lives in large complexes appear transcriptionally regulated by key subunits with short-lived transcripts [40], shorter half-life may be advantageous for circadian genes to play regulatory roles at post-translational level.

The regulation of most LCGs are dualistic in nature, i.e., they are ubiquitously expressed in multiple tissues but temporal regulated in a tissue-specific manner, crucial for the spatiotemporal gene regulation network of the circadian system [41]. Expression variations in a given developmental stage are under at least two regulation levels: tempospatial (development-specific and tissue-specific regulations) and temporal (circadian regulation). Our analyses lead to a firm conclusion that there is a higher degree of expression variations among different tissues than those across temporal phases in a given tissue (Figure 3D). In other words, the tempospatial regulation of gene expression is stronger than temporal regulation alone. This feature may reflect the fact that most of LCGs are also housekeeping genes that play essential functions and whose protein products interact with more neighbors in the protein-protein interaction network [42]. Indeed, the circadian system prefers to regulate rate-limiting housekeeping genes involved in basic biological processes [13], [32]. It is reported that the circadian system performs tissue-specific regulation at different levels. On the one hand, the circadian system may regulate tissue-specificity at transcription and post-transcription levels, such as regulating tissue-specific transcription factors [43], [44] and mRNA abundance through the interaction with microRNAs [45]–[47]. On the other hand, the circadian system may also regulate gene expressions at translation and post-translation levels, such as to control the translation process of the core clock proteins [48] and protein activation/stability through kinases [49].

At the functional level, two common features of circadian genes are obvious in the liver. First, protein folding is generally enriched in circadian genes not only found in murine liver but also in other tissues, such as brain, aorta and adipose tissue [15]. Second, the enriched protein catabolic and translation processes are partitioned into the day and the night phases, respectively. The biological processes associated with proteins are closely related to execute gene functions, and may thus be optimized for the circadian system to control rhythmical variations among tissues. It is well known that tissue-specific rhythmic is the dominant feature of circadian genes [3], [13], [50]. There are also other complications where the same genes could have diverse expressions in different phases and tissues [51]. For instance, the peak phase of the night cluster in the liver is corresponding to the reported peak feeding time of the nocturnal rodents [19], while the phase in the adrenal glands is delayed a few hours (data not shown). These tissue-specific phase shifts may be initiated from phase-specific DNA-binding rhythms of the core circadian regulators [52], [53].

Limited by the current datasets, we are not able to investigate the role of transcript splicing and to study features of circadian genes in another tissue at present time. However, RNA-seq and other applications of the next-generation sequencing technologies should be applied to circadian transcriptome studies at splice variant level [54], as well as to cover more tissue samples. A more thorough design to combine various “omics” information on circadian regulations should be more powerful for further understanding of the circadian system.

In conclusion, LCGs contain longer non-coding regions, encode smaller proteins, and show higher temporal expression variations when compared with other groups of genes, especially LNCGs. Furthermore, LCGs are orchestrated to express in four co-expression clusters with different functions. Although the majority of LCGs are ubiquitously expressed in multiple tissues with high abundance, most of them are rhythmically expressed in a tissue-specific manner. We suggest that the circadian system forms a gene regulatory network where circadian genes are selected and fine-tuned to cope with their intricate temporal and functional relationships.

## Materials and Methods

### Data used in this study

We collected all high-density temporal sampling microarray data (one hour or two hour/sample) of the murine pituitary glands [16], NIH3T3 cells and the liver under different conditions [18], [19] as well as another circadian dataset from the murine adrenal glands sampled every four hours with one replicate at each time point [55]. In addition, a dataset from a study on mRNA decay in mouse ES cells [56], one dataset about microarray-based transcriptomic data from 46 murine tissues [21] and one mass-spectrum-based proteomic data from nine murine tissues [22] are also used. Two other datasets are temporal BMAL1 binding sites list identified by Chip-Seq from mouse liver [52] and genome-wide analysis of DNA methylation level of gene promoter ranges using MeDIP-Chip in murine liver [57]. All used data are summarized in Table S4.

### Annotation of RefSeq loci and microarray probe sets

We aligned 22,315 mouse RefSeq transcripts (NCBI, May 13, 2009 update) onto the genomic sequence (UCSC, mm9) using BLAT [58], yielding 22,312 gene features. We subsequently clustered the features into loci based on sharing splicing site for multiple, overlapping, and single exons [59], and the exercise yielded 19,268 RefSeq loci, including 19,020 (98.7%) unique genes. When a locus has multiple alternatively spliced features, features with the largest number of exons and/or longest transcript were selected as representative for statistics analysis of gene parameters. The alignment of exemplar/consensus sequences of the probe sets were acquired from UCSC annotation database, and clustered into RefSeq loci. Eventually, 15,734 RefSeq loci were represented on the chip (Affymetrix MOE4302). Raw cel files of microarray data were downloaded from Gene Expression Omnibus (http://www.ncbi.nlm.nih.gov/geo/) or provided by authors, and were treated with gcRMA() function and further normalized by normalizeQuantiles() function in limma package using R software (2.10.0), then intensity values from multiple probe sets aligned to the same locus were averaged. For multiple tissues expression data (GSE10246), 116 of 182 original cDNA libraries except 66 cell line cDNA libraries were categorized into 46 unique tissues (Figure S7) based on sample origins, and the GEO accession numbers and annotated tissues were shown in Table S5. Intensity values from different cDNA libraries were further averaged according to this tissue map list. Then logarithm to base 10 of intensity value was used as expression value, and density() function in R software was used for setting the expression cut-off value which was set in the middle between non-expressed and expressed density peak [60]. We defined a RefSeq locus as expressed in a given tissue with expression value above 1.45. We further checking this cut-off by comparing liver-expressed genes with max expression value among different time points above this cut-off in time-series liver microarray data and above 0.3 RPKM in RNA-seq data [61].

### Identification of circadian and non-circadian genes

For circadian microarray data, original intensity values after normalization were analyzed by JTK_CYCLE [17] and HAYSTACK.R which was a R version of HAYSTACK [20] incorporated with p.adjust() function for calculating false discovery rate. JTK_CYCLE was used for selecting cycling probe sets firstly, and HAYSTACK.R was further used for mainly selecting non-cosine rhythmic transcripts from those probe sets omitted by JTK_CYCLE. The rhythmic transcripts identified by each method above q-value threshold were incorporated together as circadian transcripts for further analysis. The q-values set for each circadian data were shown in Table S6. For one RefSeq locus with only one rhythmic probe set identified by JTK_CYCLE or HAYSTACK, we used this rhythmic probe set for representing this locus. For one RefSeq locus with multiple rhythmic probe sets, we selected the probe set with the lowest q-value for representing this RefSeq locus. However, if more than half rhythmic probe sets of one RefSeq locus out of our period-phase filtering criterion (period difference within 4 hours and phase difference within 6 hours comparing with the representative rhythmic probe set), this RefSeq locus was not included in circadian gene list. With this method, we re-identified circadian genes in the mouse liver under *ad libitum* feeding, restricted feeding and fasting conditions, pituitary glands and NIH3T3 cells with high-density temporal sampling. Then we linked 9,066 RefSeq loci with circadian genes identified in 14 mouse tissues with low-density temporal sampling method by Yan et al. [15] through official gene symbols. For adrenal glands, we re-defined circadian genes using a similar method in analyzing high-density temporal datasets, but only selected those also in the Yan's list and then excluded the genes with fold change above 1.5 in two or more replicate time points for further analysis. At last, we collected a list of general 10,220 mouse circadian genes by combining all the circadian genes re-identified in this analysis and in the Yan's list. From the general circadian gene list, 5,825 genes that were not identified as circadian genes in liver under different feeding conditions in this analysis and Yan et al. analysis, which were named as non-liver circadian genes (NLCGs). There are remaining 9,048 RefSeq loci after excluding the general circadian gene list from the 19,268 genome RefSeq loci, which were temporally named as non-circadian genes, and those expressed (max expression values above 1.45) in the liver were defined as liver-expressed non-circadian genes (LNCGs). Through above analysis, 1,888 identified circadian genes in liver under *ad libitum* feeding (LCGs; q-value<0.001) and 1,701 LNCGs were shown in Table S1 and were used for further analysis. We estimated their false positive rates through 111 negative control genes and 104 literature-supported circadian genes (Table S2), respectively.

For drawing the heatmap of LCGs, the representative rhythmic probe sets ordered according to their phases, and intensity values of each probe set were normalized by its median. For drawing the heatmap of LNCGs, we used the average intensity values of all probe sets annotated to the same gene. Amplitude was calculated as the ratio between 95th percentile and 5th percentile intensity value of circadian representative probe set or non-circadian averaged values, respectively. Annotated GO terms of LCGs and LNCGs were analyzed on line by DAVID [62], we shown those significant enriched terms with *P*-value smaller than 0.01 and enriched fold above 2 through comparing between LCGs and LNCGs. We also selected those GO items (containing more than ten genes) special to LCGs or LNCGs with enriched *P*-value smaller than 0.01 comparing with all genome genes.

### Analyses on chromosome distribution and gene parameters of LCGs

We calculated the GC percent, gene numbers, LCGs numbers and LNCGs numbers in each autosome. We further calculated the percentage of LCGs and LNCGs to all genes in each autosome, respectively. Gene density was defined as average gene number in one megabase (Mb) in a chromosome. In a given genomic window size, each studied locus was extended half-window size of its 5′ and 3′ end at the same time. For the locus located at the 5′ or 3′ end of a chromosome, we extended one window size of its 3′ or 5′ end respectively. We further calculated the number of neighbor genes except the studied one in the new extended region. The window sizes were set 0–1.5 Mb with a step length of 15 kilobase (kb). Then we calculated and compared the average number of neighbor genes of LCGs, NLCGs, LNCGs and all gene groups. We extracted 2,049 binding sites (E-box) of BMAL1 [52] and annotated them based on their genome positions. The binding sites were annotated according to the nearest RefSeq loci by their distances to a TSS in the gene locus. If one binding site is found within 50 kb region around the TSS position of one RefSeq locus, this binding site was selected for further analysis. If multiple binding sites were in this region, we selected a representative site with highest signal and removed those redundancy sites. At the end, 1,250 annotated binding sites were sorted according to their average binding signals among different time points and the percentages of LCGs and LNCGs in top 10, 20, 50, 100, 200, 500 and all binding sites were calculated. We subsequently divided LCGs and LNCGs into four subgroups—LCGs with E-box, LCGs without E-box, LNCGs with E-box, and LNCGs without E-box, and compared the average number of their neighboring genes.

We downloaded the data genome-widely studying DNA methylation of 17,967 promoter regions and 4,566 intergenic CpG islands in the mouse liver using MeDIP coupled with 23,428 Nimblegen probe sets [57]. The genome regions covered by the microarray probe sets were primarily divided into three subgroups—strong CpG islands, weak CpG islands, CpG poor—by the authors [57]. LiftOver from UCSC was used for linking the probe positions mapped on the mm8 (NCBI36) genome to mm9 (NCBI37) genome by using mm8ToMm9.over.chain file (UCSC, Aug 5, 2010). If the center position of one probe set is localized in the 2 kb range around the TSS of its nearest RefSeq locus, we annotate this probe set to this RefSeq locus. Of the 23,428 Nimblegen probe sets, 17,066 are in the promoter regions of RefSeq loci. M-values are defined as fold changes per probe set of IP DNA (enriched) over input DNA through calculating the red (Cy5) and green (Cy3) channels as log2(IP/total) [57]. Large M-values stand for high DNA methylation levels. The percentages of LCGs and LNCGs in the strong, weak, and CpG poor groups were calculated accordingly. In addition, we compared DNA methylation of LCGs and LNCGs in the three subgroups.

CDS and 3′-UTR sequence of each RefSeq locus was extracted from its representative transcript, and genomic length was extracted from the blat result of this transcript. Then we compared the genomic, 3′-UTR and CDS length of LCGs, liver-expressed NLCGs, LNCGs and all liver-expressed genes. We downloaded all mouse mature microRNA sequences from miRBase [63] and used miRanda software (options –sc 140 –en -19) for predicting the microRNA targets [64] in the 3′-UTR sequence of each RefSeq locus. From predicted microRNA targets, we only selected those with complete alignment to the 2–8 bases (from 5′ end) of microRNA sequences and compared the number of microRNA targets between LCGs and LNCGs genes. We extracted mouse divergently-paired genes (DPGs) [65] and linked them to RefSeq loci by gene ID and gene symbol, and then selected rhythmic DPGs from liver and adrenal glands circadian genes respectively.

### Expression analysis of LCGs

For circadian microarray data, original intensity values of multiple probe sets annotated to the same RefSeq locus were averaged, and logarithms to base 10 of averaged intensity values were calculated as expression values. If one gene has the expression value above 1.45 at any one time point, we defined this gene as expressed. We sorted the liver-expressed genes according to their mean expression values among different time points (GSE11923) [18], and divided them into 28 bins with 300 genes in each bin, except the highest expression bin containing 329 genes. Then we compared the percentages of LCGs and LNCGs in each bin. We extracted the protein abundance information in nine tissues from Huttlin et al. results [22] and linked them to RefSeq loci by gene ID and gene symbol. Proteins encoded by 10,282 RefSeq loci owned protein expression abundance information. We compared the abundance (logarithms to base 2 of spectral counts) of 950 proteins encoded by LCGs, 1,109 proteins encoded by liver-expressed NLCGs, 593 proteins encoded by LNCGs and 3,834 proteins encoded by all liver-expressed genes.

We also calculated and compared mean expression values and STDs of LCGs, liver-expressed NLCGs, LNCGs and all liver-expressed genes among different tissues using 46 tissue-derived transcriptome data (GSE10246) [21], and among different time points using time sampling data in liver (GSE11923), respectively. Hierarchical cluster analysis of 46 tissues was performed using expression values of all genes presented on the chip employing hclust() function with average agglomeration method in R software. We linked mRNA half-life data from ES cells and RefSeq loci by gene symbols, and 14,663 of 19,268 RefSeq loci contained mRNA half-life information [56], and excluded those mRNA half-lives significantly (*P*-value equal or less than 0.1 by student's t-test) different between ES cells and differentiated cells. In the end, 13,518 RefSeq loci were used for studying mRNA half-lives of LCGs, liver-expressed NLCGs, LNCGs, and all liver-expressed genes in ES cells.

### Analysis on dualistic features of circadian genes

For studying expression breadth, we calculated the number of tissues where LCGs, liver-expressed NLCGs, LNCGs, and all liver-expressed genes presented on the chip was expressed to give rise to expression breadth for each gene in a 46-transcriptome datasets (GSE10246). Then we calculated the percentage of genes at each tissue expression breadth in each of these five groups, and summed the percentages in each of 11 bins, which were divided the 0–46 breadth with five numbers in each bin, except the first bin with 0 and 1, and the last two bins, one with 4 numbers from 42 to 45, and the other with the number of 46. In addition, we calculated the number of tissues where each 1,464 LCGs, 1,925 liver-expressed NLCGs, 1,190 LNCGs, 6,481 all liver-expressed or all 10,282 proteins expressed with spectral counts above zero to give the protein expression breadth, and then calculated the percentages of these five groups of proteins expressed from zero to nine tissues, respectively.

For studying tissue-specificity, we brought in high-density temporal sampling data of the pituitary [16] and NIH3T3 [18] cells. We compared the circadian genes among the liver, the pituitary, and NIH3T3 cells. We divided 1,756 liver-specific circadian genes comparing with the pituitary and NIH3T3 cells into four groups: (1) expressed only in the liver (145), (2) expressed in both the liver and the pituitary gland (129), (3) expressed in the liver and NIH3T3 cells (43), and (4) expressed in all three samples (1,439). We then selected 1,439 liver-specific circadian genes expressed in three samples and compared mean expression values and STDs among different time points of these genes in three samples.

### Analysis on temporally co-expressed LCG clusters

We grouped LCGs into clusters based on the temporal microarray data using consensusNMF.R that is a refined R script for rapidly discovering gene expression patterns based on nonnegative matrix factorization (NMF) incorporating the consensus clustering method [66]. The rank *k* range was set from two to six, and the number of clustering was set at 20. We used the consensus matrix at k = 3 from consensusNMF.R results for selecting co-expressed gene clusters with each pair having a correlation coefficient above 0.8. We selected 1,222 co-expressed genes and divided them into four main clusters from LCGs, with each cluster at least containing one hundred genes. We then re-calculated correlation coefficients of selected genes across all time points to show the order of the clusters with heatmaps. The mean phase of each cluster was named as dawn (between CT22 and CT2), day (CT2–CT10), dusk (between CT10 and CT14), and night clusters (CT14–CT22). The phase scale [0,24) was divided into 24 bins with one-hour spacing, respectively. Each phase of circadian genes was normalized by its period to ensuring its residency. We calculated the number of genes at each phase bin and showed them with radial plots. We performed expression variation and gene parameter analyses for each cluster with the methods mentioned above. We annotated GO terms of each cluster as mentioned above and selected phase-specific enriched biological processes through pair-comparisons. For example, we performed pairwise comparison among the four clusters and between LCGs and LNCGs, and we also selected those with at least three out of four pairs with *P*<0.05 and enriched fold >1.5 as phase-specific enriched GO terms. We compared the microRNA targets in one cluster with the other three clusters. The phase-specific enriched microRNAs were selected with three pairs with *P*<0.05 and enriched fold >1.5.

### Statistic methods

Statistics *P*-values were calculated by using two sample Wilcoxon test (the wilcox.test() function in R software), where a one-side alternative hypothesis was set. We showed “*P*<2.2e-16” when *P*-values were smaller than 2.2e-16, but without reporting the exact *P*-values. The cor.test() function was used for calculating statistics *P*-values of correlation between the percentage of LCGs or LNCGs in each autosome and autosome GC content. Pearson's chi-squared test (the chisq.test () function in R software) and Fisher's exact test (the fisher.test () function in R software) were used for calculating statistics *P*-values of enrichment of GO terms or microRNAs.

## Supporting Information

Figure S1 **Comparison of liver-associated genes identified by microarray and RNA-seq.** (A) Density plot of expression values of all RefSeq loci presented on the microarray based on high-density temporal sampling of the liver (GSE11923). (B) Venn diagram shows the overlap of liver-associated genes identified from the microarrays (GSE11923, max expression value above 1.45) and RNA-seq (RPKM above 0.3).
(TIF)

Figure S2 **Enriched GO terms in the LCGs and LNCGs.** Functional categories of LCGs (red) and LNCGs (blue) are annotated based on Gene Ontology (GO) analyzed using DAVID. Enriched functional terms are shown with enriched fold between the gene groups. Enriched GO terms in LCGs and LNCGs are shown in red (A) and blue (B), respectively. Red and blue triangle indicates the GO term is specially annotated to LCGs and LNCGs, respectively.
(TIF)

Figure S3 **The percentage of genes containing E-box in LCGs and LNCGs, and comparison the number of neighbor genes among LCGs/LNCGs with/without E-box.** (A) The histogram shows the percentages of LCGs (red) and LNCGs (blue) genes for each BMAL1 binding site bins (from top 10 sites to all sites), which are ranked according to their mean binding signals among different time points. (B) The average numbers of neighboring genes for LCGs with E-box (red), LCGs without E-box (orange), LNCGs with E-box (green) and LNCGs without E-box (blue) are calculated in a given genomic length window (from zero to 1.5 Mb with a step length of 15 kb).
(TIF)

Figure S4 **DNA methylation of LCGs and LNCGs.** (A) The histogram shows the percentages of genes with strong, weak and poor CpG islands in LCGs (red) and LNCGs (blue), respectively. (B) DNA methylation levels of promoter regions of LCGs (red) and LNCGs (blue) in strong, weak and poor CpG island subgroups, respectively. M-values are calculated as fold changes per probe set of enriched methylated DNA over input DNA, and the large M-value indicates high DNA methylation level. The boxes depict data between the 25th and 75th percentiles with central horizontal lines and solid circles representing the median and mean values, respectively, and with whiskers showing the 5th and 95th percentiles. *P*-values are calculated based on the Wilcoxon rank sum test. Strong, weak, and poor stand for strong, weak and poor CpG islands, respectively. (**), *P*-value<0.01. (***), *P*-value<0.001. NS, not significant.
(TIF)

Figure S5 **Predicted microRNA targets in LCGs and LNCGs, and enriched microRNAs in day and dusk cluster.** (A) The number (using logarithm to base 2) of microRNA targets predicted in LCGs (red) and LNCGs (blue) are shown in a box plot. The boxes depict data between the 25th and 75th percentiles with central horizontal lines and solid circles representing the median and mean values, respectively, and with whiskers showing the 5th and 95th percentiles. *P*-values are calculated based on the Wilcoxon rank sum test. (**), *P*-value<0.01. (B) The histogram shows the relative enriched ratios of predicted microRNA (mmu-miR-1187) targets in dawn (purple), day (orange), dusk (green), and night cluster (blue) comparing with day cluster. (C) The histogram shows the relative enriched ratios of predicted microRNA targets in dawn (purple), day (orange), dusk (green) and night clusters (blue) comparing with dusk cluster. The purple line indicates that there is no predicted target of microRNA (mmu-miR-466d-3p) in dawn cluster.
(TIF)

Figure S6 **Enriched biological processes in dawn, day, and night circadian clusters.** Histograms show the relative enriched ratios of biological processes in dawn (purple), day (orange), dusk (green), night cluster (blue), and LNCGs (grey) as compared with dawn (A), day (B), and night (C) cluster. The color line indicates that there is no gene annotated to the biological process in the corresponding group.
(TIF)

Figure S7 **A dendrogram of genes from 46 tissues clustered based on all RefSeq loci presenting on microarrays (GSE10246).**
(TIF)

Table S1 **LCGs and LNCGs with their gene parameters.**
(XLS)

Table S2 **Mouse liver circadian, non-circadian, and unexpressed genes extracted from literature.**
(XLS)

Table S3 **Divergently-paired circadian genes in mouse liver and adrenal glands.**
(XLS)

Table S4 **Data used in this analysis and associated references.**
(DOC)

Table S5 **GEO accession numbers and their corresponding annotated tissues.**
(XLS)

Table S6 **Circadian gene numbers and associated q-value cut-off from temporal microarray data.**
(XLS)

## References

[1] Konopka RJ, Benzer S (1971) Clock mutants of Drosophila melanogaster. Proc Natl Acad Sci U S A 68: 2112–2116. 5002428 10.1073/pnas.68.9.2112 PMC389363

[2] Pittendrigh CS (1993) Temporal organization: reflections of a Darwinian clock-watcher. Annu Rev Physiol 55: 16–54. 8466172 10.1146/annurev.ph.55.030193.000313

[3] Doherty CJ, Kay SA (2010) Circadian control of global gene expression patterns. Annu Rev Genet 44: 419–444. 20809800 10.1146/annurev-genet-102209-163432 PMC4251774

[4] Hogenesch JB, Ueda HR (2011) Understanding systems-level properties: timely stories from the study of clocks. Nat Rev Genet 12: 407–416. 21556016 10.1038/nrg2972

[5] Ralph MR, Foster RG, Davis FC, Menaker M (1990) Transplanted suprachiasmatic nucleus determines circadian period. Science 247: 975–978. 2305266 10.1126/science.2305266

[6] Reppert SM, Weaver DR (2001) Molecular analysis of mammalian circadian rhythms. Annu Rev Physiol 63: 647–676. 11181971 10.1146/annurev.physiol.63.1.647

[7] Dibner C, Schibler U, Albrecht U (2010) The mammalian circadian timing system: organization and coordination of central and peripheral clocks. Annu Rev Physiol 72: 517–549. 20148687 10.1146/annurev-physiol-021909-135821

[8] Takahashi JS, Hong HK, Ko CH, McDearmon EL (2008) The genetics of mammalian circadian order and disorder: implications for physiology and disease. Nat Rev Genet 9: 764–775. 18802415 10.1038/nrg2430 PMC3758473

[9] Ko CH, Takahashi JS (2006) Molecular components of the mammalian circadian clock. Hum Mol Genet 15 Spec No 2: R271–277. 16987893 10.1093/hmg/ddl207

[10] Ueda HR, Hayashi S, Chen W, Sano M, Machida M, et al (2005) System-level identification of transcriptional circuits underlying mammalian circadian clocks. Nat Genet 37: 187–192. 15665827 10.1038/ng1504

[11] Harrisingh MC, Nitabach MN (2008) Circadian rhythms. Integrating circadian timekeeping with cellular physiology. Science 320: 879–880. 18487177 10.1126/science.1158619

[12] Storch KF, Lipan O, Leykin I, Viswanathan N, Davis FC, et al (2002) Extensive and divergent circadian gene expression in liver and heart. Nature 417: 78–83. 11967526 10.1038/nature744

[13] Panda S, Antoch MP, Miller BH, Su AI, Schook AB, et al (2002) Coordinated transcription of key pathways in the mouse by the circadian clock. Cell 109: 307–320. 12015981 10.1016/s0092-8674(02)00722-5

[14] Ptitsyn AA, Gimble JM (2011) True or false: all genes are rhythmic. Ann Med 43: 1–12. 21142579 10.3109/07853890.2010.538078

[15] Yan J, Wang H, Liu Y, Shao C (2008) Analysis of gene regulatory networks in the mammalian circadian rhythm. PLoS Comput Biol 4: e1000193. 18846204 10.1371/journal.pcbi.1000193 PMC2543109

[16] Hughes M, Deharo L, Pulivarthy SR, Gu J, Hayes K, et al (2007) High-resolution time course analysis of gene expression from pituitary. Cold Spring Harb Symp Quant Biol 72: 381–386. 18419295 10.1101/sqb.2007.72.011 PMC2670782

[17] Hughes ME, Hogenesch JB, Kornacker K (2010) JTK_CYCLE: an efficient nonparametric algorithm for detecting rhythmic components in genome-scale data sets. J Biol Rhythms 25: 372–380. 20876817 10.1177/0748730410379711 PMC3119870

[18] Hughes ME, DiTacchio L, Hayes KR, Vollmers C, Pulivarthy S, et al (2009) Harmonics of circadian gene transcription in mammals. PLoS Genet 5: e1000442. 19343201 10.1371/journal.pgen.1000442 PMC2654964

[19] Vollmers C, Gill S, DiTacchio L, Pulivarthy SR, Le HD, et al (2009) Time of feeding and the intrinsic circadian clock drive rhythms in hepatic gene expression. Proc Natl Acad Sci U S A 106: 21453–21458. 19940241 10.1073/pnas.0909591106 PMC2795502

[20] Michael TP, Mockler TC, Breton G, McEntee C, Byer A, et al (2008) Network discovery pipeline elucidates conserved time-of-day-specific cis-regulatory modules. PLoS Genet 4: e14. 18248097 10.1371/journal.pgen.0040014 PMC2222925

[21] Lattin JE, Schroder K, Su AI, Walker JR, Zhang J, et al (2008) Expression analysis of G Protein-Coupled Receptors in mouse macrophages. Immunome Res 4: 5. 18442421 10.1186/1745-7580-4-5 PMC2394514

[22] Huttlin EL, Jedrychowski MP, Elias JE, Goswami T, Rad R, et al (2010) A tissue-specific atlas of mouse protein phosphorylation and expression. Cell 143: 1174–1189. 21183079 10.1016/j.cell.2010.12.001 PMC3035969

[23] Stokkan KA, Yamazaki S, Tei H, Sakaki Y, Menaker M (2001) Entrainment of the circadian clock in the liver by feeding. Science 291: 490–493. 11161204 10.1126/science.291.5503.490

[24] Lamia KA, Storch KF, Weitz CJ (2008) Physiological significance of a peripheral tissue circadian clock. Proc Natl Acad Sci U S A 105: 15172–15177. 18779586 10.1073/pnas.0806717105 PMC2532700

[25] Turek FW, Joshu C, Kohsaka A, Lin E, Ivanova G, et al (2005) Obesity and metabolic syndrome in circadian Clock mutant mice. Science 308: 1043–1045. 15845877 10.1126/science.1108750 PMC3764501

[26] Hurst LD, Pal C, Lercher MJ (2004) The evolutionary dynamics of eukaryotic gene order. Nat Rev Genet 5: 299–310. 15131653 10.1038/nrg1319

[27] Versteeg R, van Schaik BD, van Batenburg MF, Roos M, Monajemi R, et al (2003) The human transcriptome map reveals extremes in gene density, intron length, GC content, and repeat pattern for domains of highly and weakly expressed genes. Genome Res 13: 1998–2004. 12915492 10.1101/gr.1649303 PMC403669

[28] Hazen SP, Naef F, Quisel T, Gendron JM, Chen H, et al (2009) Exploring the transcriptional landscape of plant circadian rhythms using genome tiling arrays. Genome Biol 10: R17. 19210792 10.1186/gb-2009-10-2-r17 PMC2688271

[29] Schwanhausser B, Busse D, Li N, Dittmar G, Schuchhardt J, et al (2011) Global quantification of mammalian gene expression control. Nature 473: 337–342. 21593866 10.1038/nature10098

[30] Vogel C, Abreu Rde S, Ko D, Le SY, Shapiro BA, et al (2010) Sequence signatures and mRNA concentration can explain two-thirds of protein abundance variation in a human cell line. Mol Syst Biol 6: 400. 20739923 10.1038/msb.2010.59 PMC2947365

[31] Lackner DH, Beilharz TH, Marguerat S, Mata J, Watt S, et al (2007) A network of multiple regulatory layers shapes gene expression in fission yeast. Mol Cell 26: 145–155. 17434133 10.1016/j.molcel.2007.03.002 PMC1885965

[32] Reddy AB, Karp NA, Maywood ES, Sage EA, Deery M, et al (2006) Circadian orchestration of the hepatic proteome. Curr Biol 16: 1107–1115. 16753565 10.1016/j.cub.2006.04.026

[33] Raj A, van Oudenaarden A (2008) Nature, nurture, or chance: stochastic gene expression and its consequences. Cell 135: 216–226. 18957198 10.1016/j.cell.2008.09.050 PMC3118044

[34] Zhang EE, Liu AC, Hirota T, Miraglia LJ, Welch G, et al (2009) A genome-wide RNAi screen for modifiers of the circadian clock in human cells. Cell 139: 199–210. 19765810 10.1016/j.cell.2009.08.031 PMC2777987

[35] Vitaterna MH, Ko CH, Chang AM, Buhr ED, Fruechte EM, et al (2006) The mouse Clock mutation reduces circadian pacemaker amplitude and enhances efficacy of resetting stimuli and phase-response curve amplitude. Proc Natl Acad Sci U S A 103: 9327–9332. 16754844 10.1073/pnas.0603601103 PMC1474012

[36] Rabani M, Levin JZ, Fan L, Adiconis X, Raychowdhury R, et al (2011) Metabolic labeling of RNA uncovers principles of RNA production and degradation dynamics in mammalian cells. Nat Biotechnol 29: 436–442. 21516085 10.1038/nbt.1861 PMC3114636

[37] Yagita K, Horie K, Koinuma S, Nakamura W, Yamanaka I, et al (2010) Development of the circadian oscillator during differentiation of mouse embryonic stem cells in vitro. Proc Natl Acad Sci U S A 107: 3846–3851. 20133594 10.1073/pnas.0913256107 PMC2840478

[38] Dolatshad H, Cary AJ, Davis FC (2010) Differential expression of the circadian clock in maternal and embryonic tissues of mice. PLoS One 5: e9855. 20352049 10.1371/journal.pone.0009855 PMC2844431

[39] Yamazaki S, Yoshikawa T, Biscoe EW, Numano R, Gallaspy LM, et al (2009) Ontogeny of circadian organization in the rat. J Biol Rhythms 24: 55–63. 19150929 10.1177/0748730408328438 PMC2665126

[40] Friedel CC, Dolken L, Ruzsics Z, Koszinowski UH, Zimmer R (2009) Conserved principles of mammalian transcriptional regulation revealed by RNA half-life. Nucleic Acids Res 37: e115. 19561200 10.1093/nar/gkp542 PMC2761256

[41] Kholodenko BN, Hancock JF, Kolch W (2010) Signalling ballet in space and time. Nat Rev Mol Cell Biol 11: 414–426. 20495582 10.1038/nrm2901 PMC2977972

[42] Lin WH, Liu WC, Hwang MJ (2009) Topological and organizational properties of the products of house-keeping and tissue-specific genes in protein-protein interaction networks. BMC Syst Biol 3: 32. 19284572 10.1186/1752-0509-3-32 PMC2663781

[43] Masri S, Sassone-Corsi P (2010) Plasticity and specificity of the circadian epigenome. Nat Neurosci 13: 1324–1329. 20975756 10.1038/nn.2668 PMC4071955

[44] Feng D, Liu T, Sun Z, Bugge A, Mullican SE, et al (2011) A circadian rhythm orchestrated by histone deacetylase 3 controls hepatic lipid metabolism. Science 331: 1315–1319. 21393543 10.1126/science.1198125 PMC3389392

[45] Gatfield D, Le Martelot G, Vejnar CE, Gerlach D, Schaad O, et al (2009) Integration of microRNA miR-122 in hepatic circadian gene expression. Genes Dev 23: 1313–1326. 19487572 10.1101/gad.1781009 PMC2701584

[46] Cheng HY, Papp JW, Varlamova O, Dziema H, Russell B, et al (2007) microRNA modulation of circadian-clock period and entrainment. Neuron 54: 813–829. 17553428 10.1016/j.neuron.2007.05.017 PMC2590749

[47] Kojima S, Gatfield D, Esau CC, Green CB (2010) MicroRNA-122 modulates the rhythmic expression profile of the circadian deadenylase Nocturnin in mouse liver. PLoS One 5: e11264. 20582318 10.1371/journal.pone.0011264 PMC2889834

[48] Kim DY, Woo KC, Lee KH, Kim TD, Kim KT (2010) hnRNP Q and PTB modulate the circadian oscillation of mouse Rev-erb alpha via IRES-mediated translation. Nucleic Acids Res 38: 7068–7078. 20576698 10.1093/nar/gkq569 PMC2978350

[49] Lee C, Etchegaray JP, Cagampang FR, Loudon AS, Reppert SM (2001) Posttranslational mechanisms regulate the mammalian circadian clock. Cell 107: 855–867. 11779462 10.1016/s0092-8674(01)00610-9

[50] Zvonic S, Ptitsyn AA, Conrad SA, Scott LK, Floyd ZE, et al (2006) Characterization of peripheral circadian clocks in adipose tissues. Diabetes 55: 962–970. 16567517 10.2337/diabetes.55.04.06.db05-0873

[51] Ueda HR, Chen W, Adachi A, Wakamatsu H, Hayashi S, et al (2002) A transcription factor response element for gene expression during circadian night. Nature 418: 534–539. 12152080 10.1038/nature00906

[52] Rey G, Cesbron F, Rougemont J, Reinke H, Brunner M, et al (2011) Genome-Wide and Phase-Specific DNA-Binding Rhythms of BMAL1 Control Circadian Output Functions in Mouse Liver. PLoS Biol 9: e1000595. 21364973 10.1371/journal.pbio.1000595 PMC3043000

[53] Bozek K, Relogio A, Kielbasa SM, Heine M, Dame C, et al (2009) Regulation of clock-controlled genes in mammals. PLoS One 4: e4882. 19287494 10.1371/journal.pone.0004882 PMC2654074

[54] Hughes ME, Grant GR, Paquin C, Qian J, Nitabach MN (2012) Deep sequencing the circadian and diurnal transcriptome of Drosophila brain. Genome Res 22: 1266–1281. 22472103 10.1101/gr.128876.111 PMC3396368

[55] Oster H, Damerow S, Hut RA, Eichele G (2006) Transcriptional profiling in the adrenal gland reveals circadian regulation of hormone biosynthesis genes and nucleosome assembly genes. J Biol Rhythms 21: 350–361. 16998155 10.1177/0748730406293053

[56] Sharova LV, Sharov AA, Nedorezov T, Piao Y, Shaik N, et al (2009) Database for mRNA half-life of 19 977 genes obtained by DNA microarray analysis of pluripotent and differentiating mouse embryonic stem cells. DNA Res 16: 45–58. 19001483 10.1093/dnares/dsn030 PMC2644350

[57] Lempiainen H, Muller A, Brasa S, Teo SS, Roloff TC, et al (2011) Phenobarbital mediates an epigenetic switch at the constitutive androstane receptor (CAR) target gene Cyp2b10 in the liver of B6C3F1 mice. PLoS One 6: e18216. 21455306 10.1371/journal.pone.0018216 PMC3063791

[58] Kent WJ (2002) BLAT–the BLAST-like alignment tool. Genome Res 12: 656–664. 11932250 10.1101/gr.229202 PMC187518

[59] Zhu J, He F, Song S, Wang J, Yu J (2008) How many human genes can be defined as housekeeping with current expression data? BMC Genomics 9: 172. 18416810 10.1186/1471-2164-9-172 PMC2396180

[60] Hebenstreit D, Fang M, Gu M, Charoensawan V, van Oudenaarden A, et al (2011) RNA sequencing reveals two major classes of gene expression levels in metazoan cells. Mol Syst Biol 7: 497. 21654674 10.1038/msb.2011.28 PMC3159973

[61] Mortazavi A, Williams BA, McCue K, Schaeffer L, Wold B (2008) Mapping and quantifying mammalian transcriptomes by RNA-seq. Nat Methods 5: 621–628. 18516045 10.1038/nmeth.1226 PMC13303166

[62] Huang da W, Sherman BT, Lempicki RA (2009) Systematic and integrative analysis of large gene lists using DAVID bioinformatics resources. Nat Protoc 4: 44–57. 19131956 10.1038/nprot.2008.211

[63] Griffiths-Jones S, Grocock RJ, van Dongen S, Bateman A, Enright AJ (2006) miRBase: microRNA sequences, targets and gene nomenclature. Nucleic Acids Res 34: D140–144. 16381832 10.1093/nar/gkj112 PMC1347474

[64] Enright AJ, John B, Gaul U, Tuschl T, Sander C, et al (2003) MicroRNA targets in Drosophila. Genome Biol 5: R1. 14709173 10.1186/gb-2003-5-1-r1 PMC395733

[65] Yang L, Yu J (2009) A comparative analysis of divergently-paired genes (DPGs) among Drosophila and vertebrate genomes. BMC Evol Biol 9: 55. 19284596 10.1186/1471-2148-9-55 PMC2670823

[66] Brunet JP, Tamayo P, Golub TR, Mesirov JP (2004) Metagenes and molecular pattern discovery using matrix factorization. Proc Natl Acad Sci U S A 101: 4164–4169. 15016911 10.1073/pnas.0308531101 PMC384712

