# Supplementary figures and images for: Gene and Genome Parameters of Mammalian Liver Circadian Genes (LCGs)
Source: PLoS One. 2012 Oct 10;7(10):e46961. doi: 10.1371/journal.pone.0046961 (PMC3468600; doi:10.1371/journal.pone.0046961)

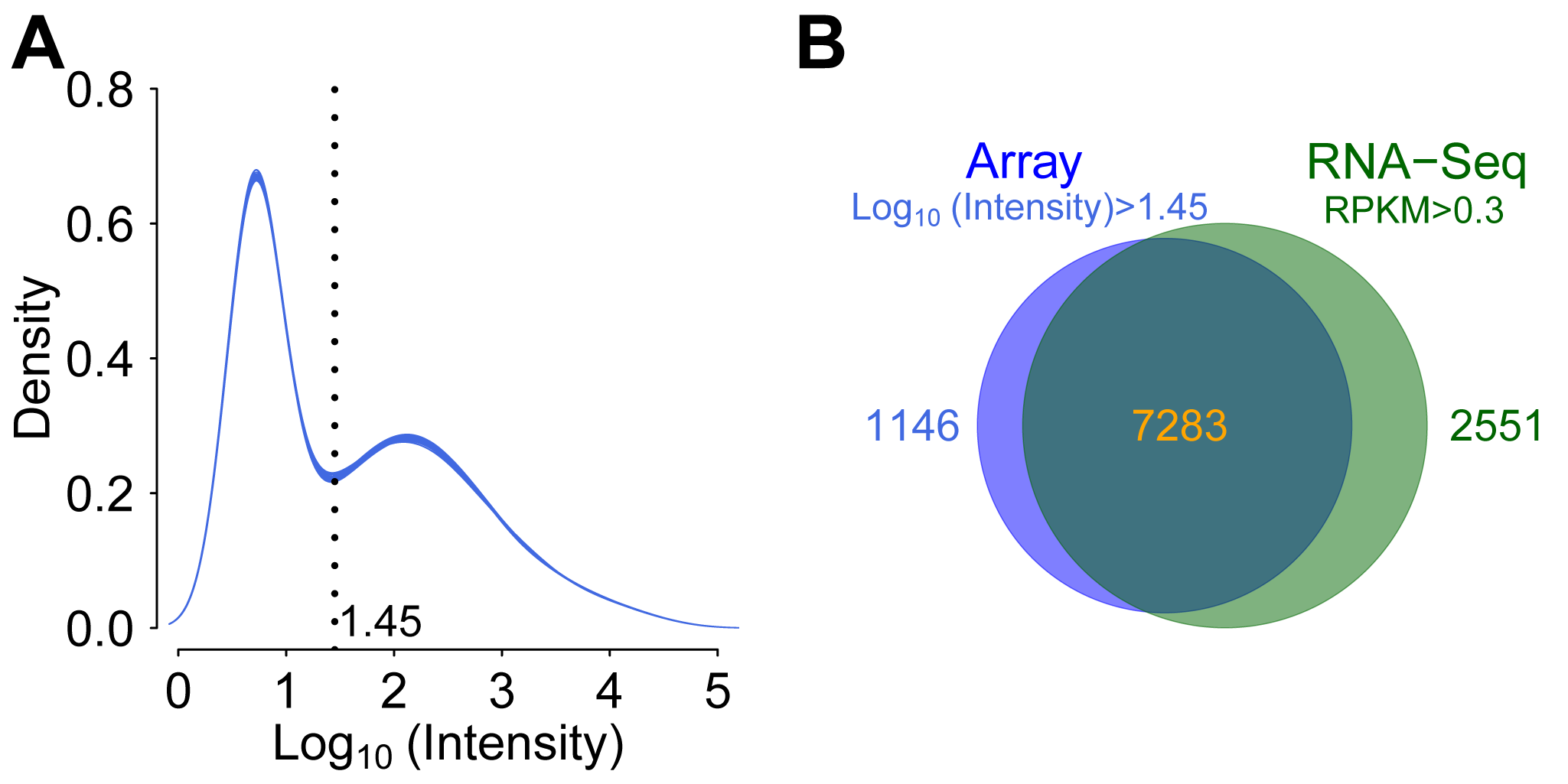

Supplement: Figure S1 — Comparison of liver-associated genes identified by microarray and RNA-seq. (A) Density plot of expression values of all RefSeq loci presented on the microarray based on high-density temporal sampling of the liver (GSE11923). (B) Venn diagram shows the overlap of liver-associated genes identified from the microarrays (GSE11923, max expression value above 1.45) and RNA-seq (RPKM above 0.3). (TIF) [file pone.0046961.s001.tif]

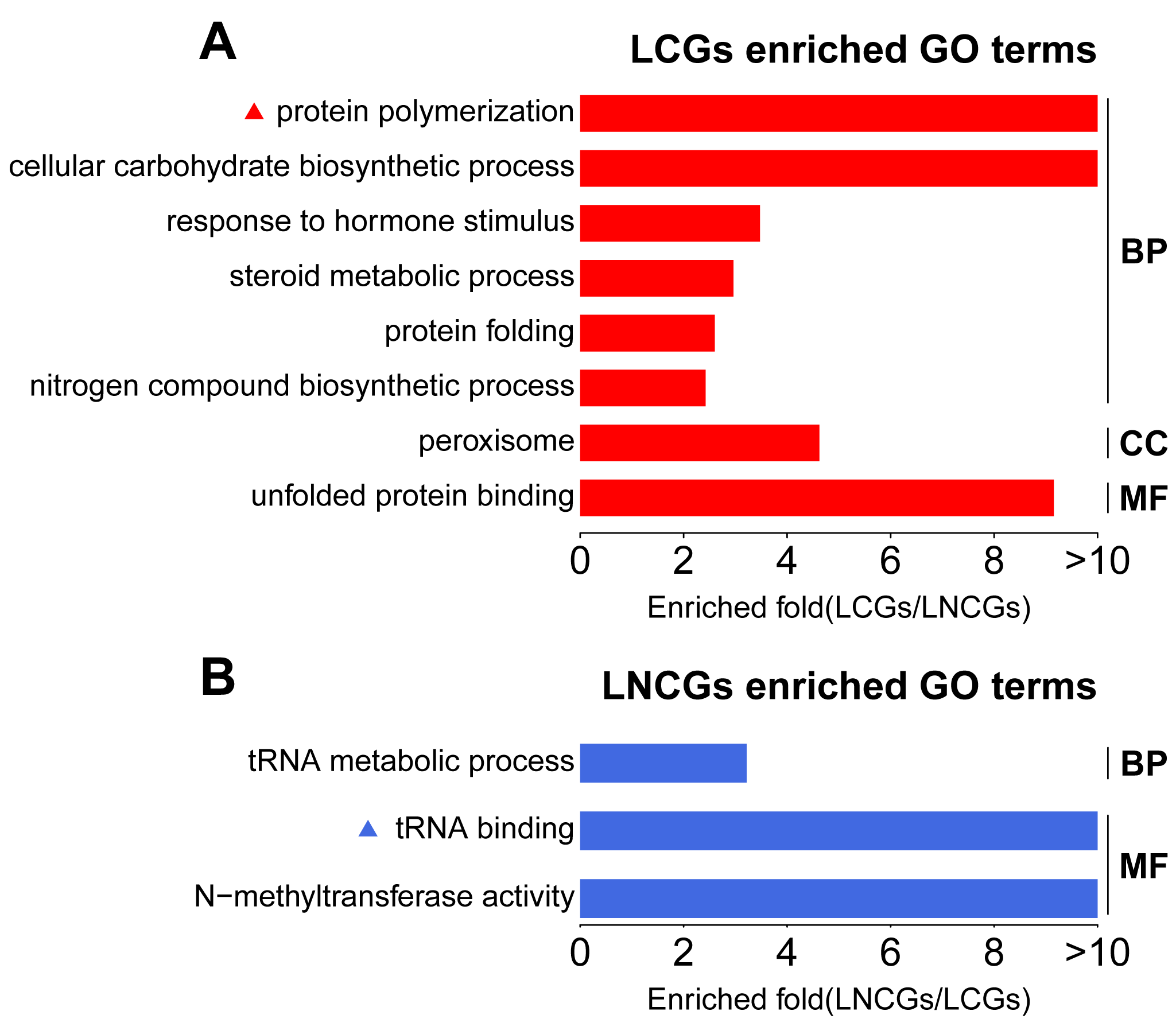

Supplement: Figure S2 — Enriched GO terms in the LCGs and LNCGs. Functional categories of LCGs (red) and LNCGs (blue) are annotated based on Gene Ontology (GO) analyzed using DAVID. Enriched functional terms are shown with enriched fold between the gene groups. Enriched GO terms in LCGs and LNCGs are shown in red (A) and blue (B), respectively. Red and blue triangle indicates the GO term is specially annotated to LCGs and LNCGs, respectively. (TIF) [file pone.0046961.s002.tif]

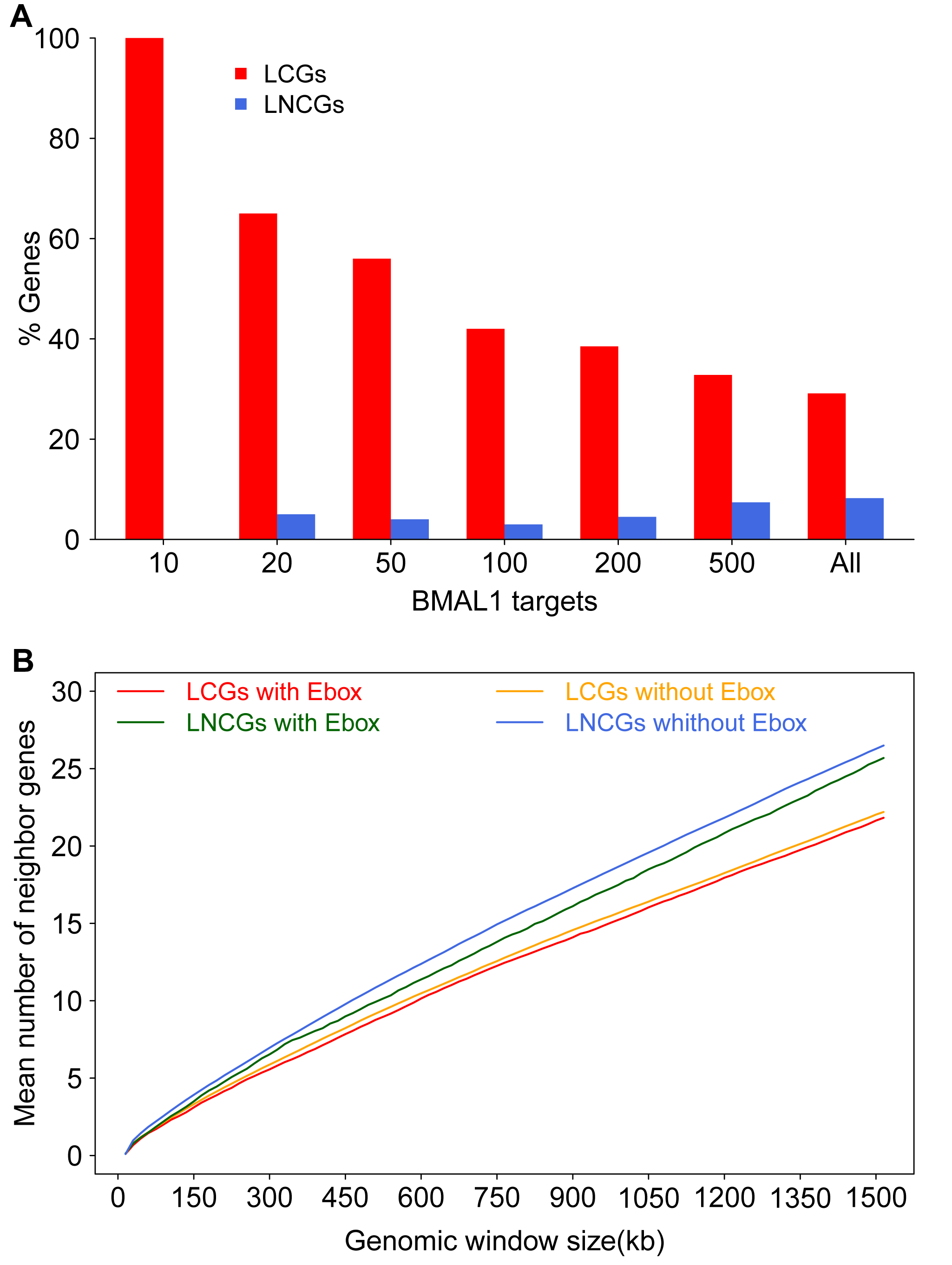

Supplement: Figure S3 — The percentage of genes containing E-box in LCGs and LNCGs, and comparison the number of neighbor genes among LCGs/LNCGs with/without E-box. (A) The histogram shows the percentages of LCGs (red) and LNCGs (blue) genes for each BMAL1 binding site bins (from top 10 sites to all sites), which are ranked according to their mean binding signals among different time points. (B) The average numbers of neighboring genes for LCGs with E-box (red), LCGs without E-box (orange), LNCGs with E-box (green) and LNCGs without E-box (blue) are calculated in a given genomic length window (from zero to 1.5 Mb with a step length of 15 kb). (TIF) [file pone.0046961.s003.tif]

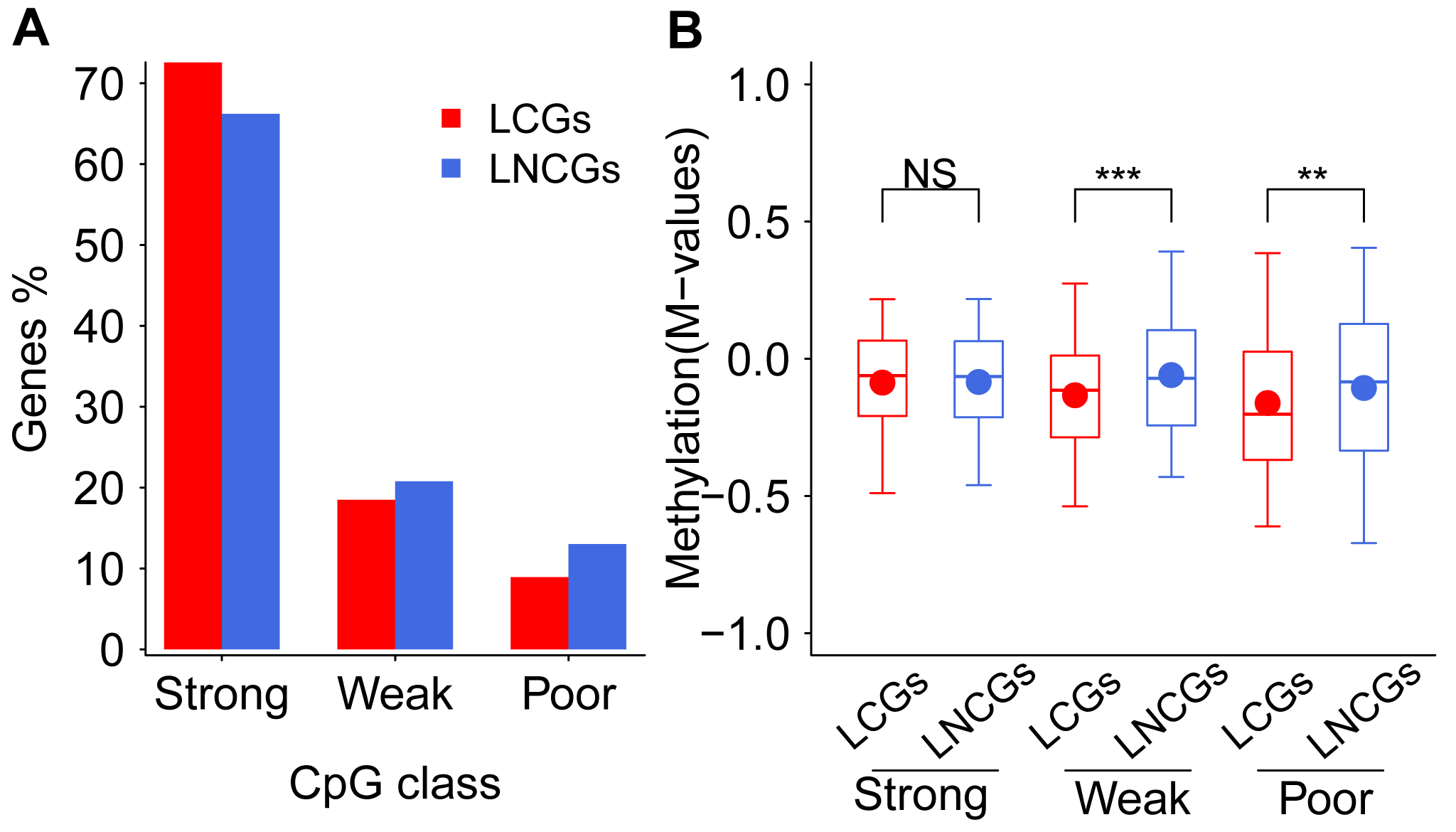

Supplement: Figure S4 — DNA methylation of LCGs and LNCGs. (A) The histogram shows the percentages of genes with strong, weak and poor CpG islands in LCGs (red) and LNCGs (blue), respectively. (B) DNA methylation levels of promoter regions of LCGs (red) and LNCGs (blue) in strong, weak and poor CpG island subgroups, respectively. M-values are calculated as fold changes per probe set of enriched methylated DNA over input DNA, and the large M-value indicates high DNA methylation level. The boxes depict data between the 25th and 75th percentiles with central horizontal lines and solid circles representing the median and mean values, respectively, and with whiskers showing the 5th and 95th percentiles. P-values are calculated based on the Wilcoxon rank sum test. Strong, weak, and poor stand for strong, weak and poor CpG islands, respectively. (**), P-value<0.01. (***), P-value<0.001. NS, not significant. (TIF) [file pone.0046961.s004.tif]

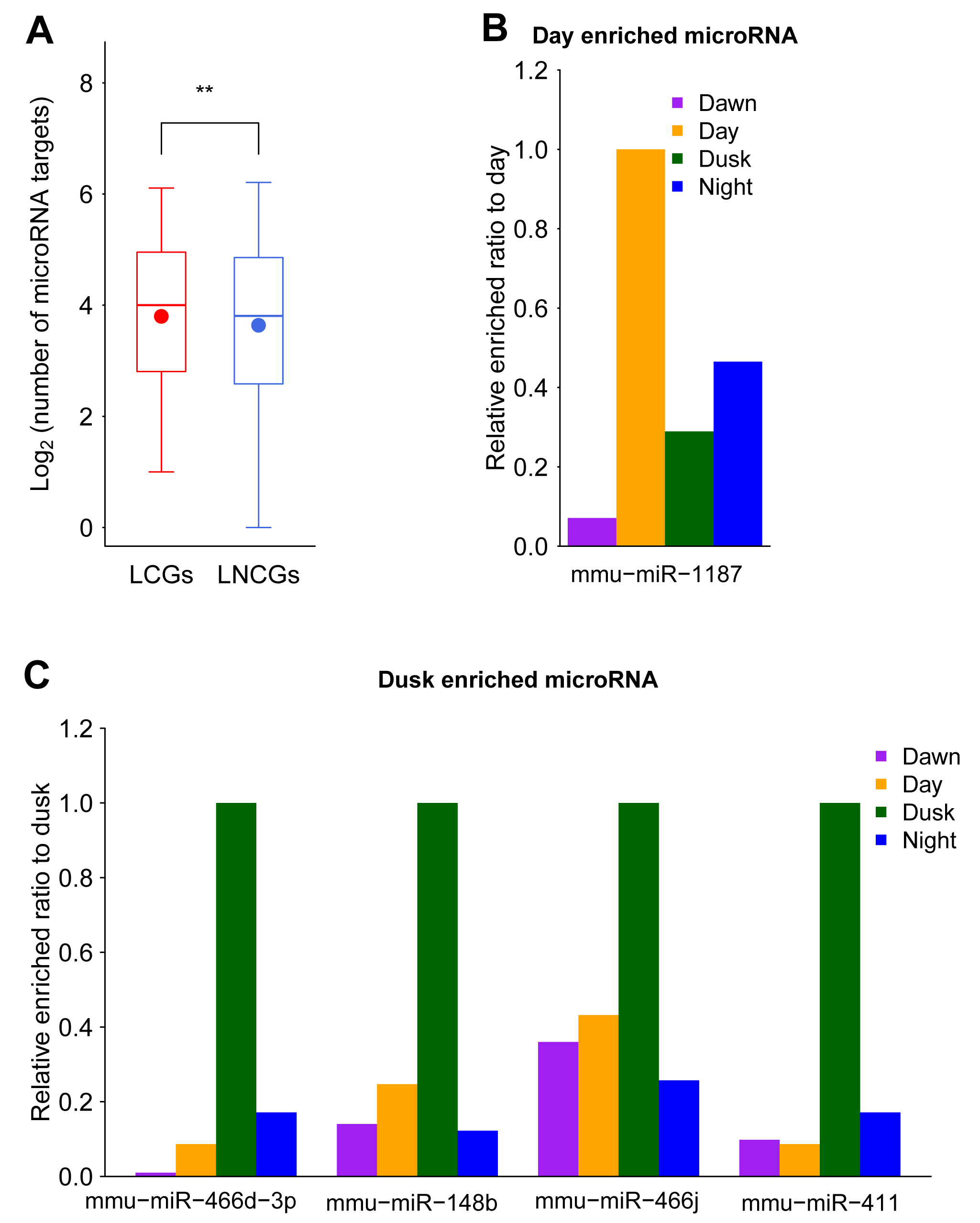

Supplement: Figure S5 — Predicted microRNA targets in LCGs and LNCGs, and enriched microRNAs in day and dusk cluster. (A) The number (using logarithm to base 2) of microRNA targets predicted in LCGs (red) and LNCGs (blue) are shown in a box plot. The boxes depict data between the 25th and 75th percentiles with central horizontal lines and solid circles representing the median and mean values, respectively, and with whiskers showing the 5th and 95th percentiles. P-values are calculated based on the Wilcoxon rank sum test. (**), P-value<0.01. (B) The histogram shows the relative enriched ratios of predicted microRNA (mmu-miR-1187) targets in dawn (purple), day (orange), dusk (green), and night cluster (blue) comparing with day cluster. (C) The histogram shows the relative enriched ratios of predicted microRNA targets in dawn (purple), day (orange), dusk (green) and night clusters (blue) comparing with dusk cluster. The purple line indicates that there is no predicted target of microRNA (mmu-miR-466d-3p) in dawn cluster. (TIF) [file pone.0046961.s005.tif]

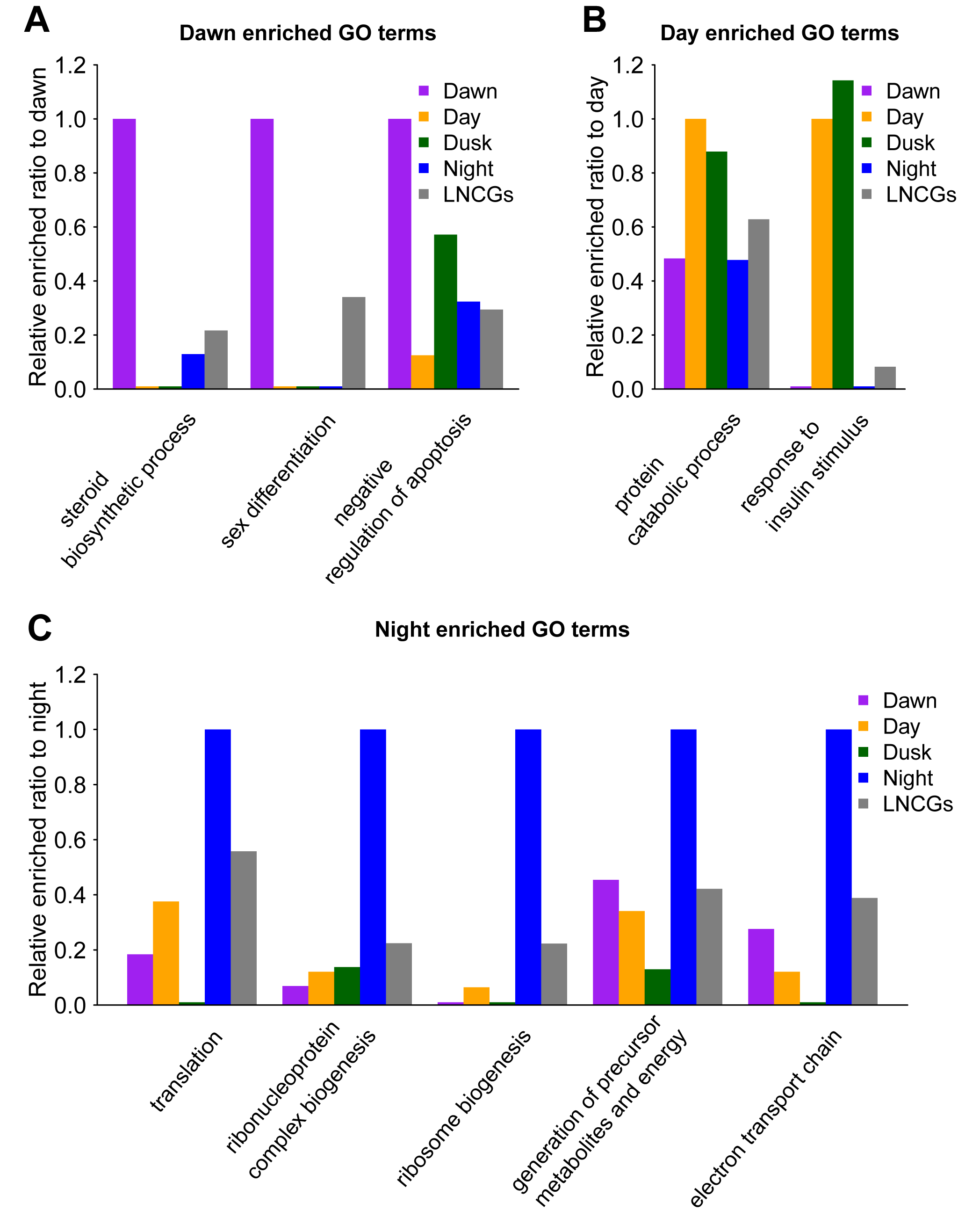

Supplement: Figure S6 — Enriched biological processes in dawn, day, and night circadian clusters. Histograms show the relative enriched ratios of biological processes in dawn (purple), day (orange), dusk (green), night cluster (blue), and LNCGs (grey) as compared with dawn (A), day (B), and night (C) cluster. The color line indicates that there is no gene annotated to the biological process in the corresponding group. (TIF) [file pone.0046961.s006.tif]

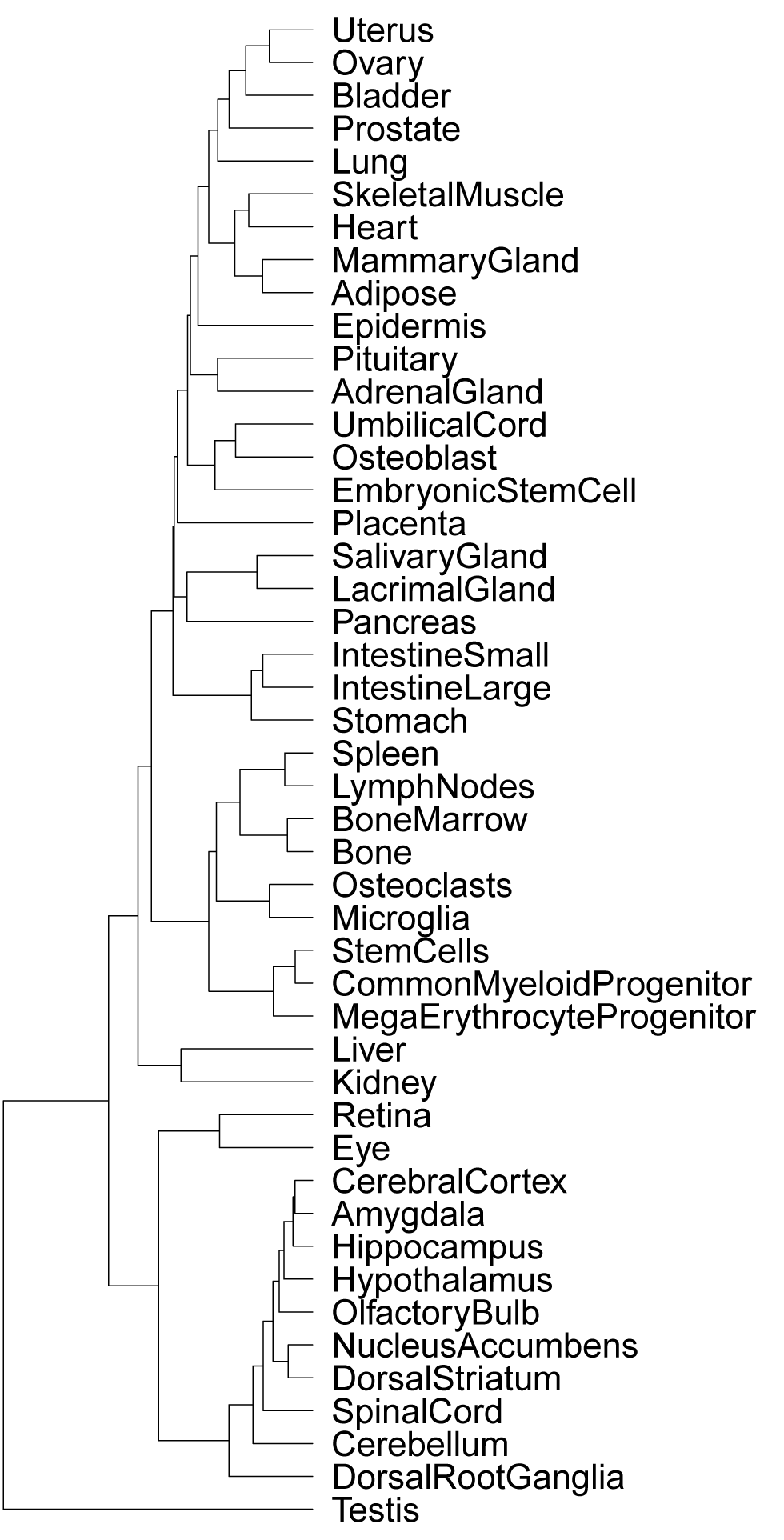

Supplement: Figure S7 — A dendrogram of genes from 46 tissues clustered based on all RefSeq loci presenting on microarrays (GSE10246). (TIF) [file pone.0046961.s007.tif]
