# Supplementary material for: Gene and Genome Parameters of Mammalian Liver Circadian Genes (LCGs)
Source: PLoS One. 2012 Oct 10;7(10):e46961. doi: 10.1371/journal.pone.0046961 (PMC3468600; doi:10.1371/journal.pone.0046961)
Supplement: Table S4 — Data used in this analysis and associated references. (DOC) [file pone.0046961.s011.doc]

**Table S4**. Data list used in analysis

| **Reference** | **Tissue** | **Technique Platform** | **Data Type** | **Time Series** | **Data Source** |
| --- | --- | --- | --- | --- | --- |
| Hughes et al. 2009 [1] | Mouse (C57BL/6J) liver | Microarray Affymetrix MOE 4302 | Raw cel files | Every 1 hour between CT18 and CT62 | GSE11923 |
| Vollmers et al. 2009 [2] | Mouse (C57BL/6J) liver | Microarray Affymetrix MOE 4302 | Raw cel files | Every 1 hour between CT24 and CT47 under temporally restricted feeding; Every 2 hours between CT 2 and CT46 under fasting | GSE13093 |
| Hughes et al. 2007 [3] | Mouse (C57BL/6J) Pituitary | Microarray Affymetrix MOE 4302 | Raw cel files | Every 1 hour between CT18 and CT62 | Author provided |
| Hughes et al. 2009 [1] | NIH3T3 cell line | Microarray Affymetrix MOE 4302 | Raw cel files | Every 1 hour between CT18 and CT62 | GSE11922 |
| Oster et al. 2006 [4] | Mouse (C57BL/6J) adrenal gland | Microarray Affymetrix MOE 4302 | Raw cel files | Every 4 hours between CT2 and CT46 with one replicate at each time point | Author provided |
| Sharova et al. 2009 [5] | ES cell line (C57BL/6J and 129S6- SvEvTac) | Microarray Agilent | mRNA decay rate file | Cells were harvested at 0, 1, 2, 4 and 8 hour after addition of actinomycin D | Extracted from literarure |
| Huttlin et al. 2010 [6] | Mouse (Swiss Webster) 9 tissues | LC-MS/MS | Protein list | 6 hours after lights turned on | Author provided |
| Lattin et al. 2008 [7] | Mouse (C57BL/6J) 46 tissues | Microarray Affymetrix MOE 4302 | Raw cel files | NA | GSE10246 |
| Rey et al. 2011 [8] | Mouse liver | Chip-Seq | BMAL1 binding sites in genome | Every 4 hours between ZT2 and ZT22 with one replicate at each time point | Extracted from literarure |
| Lempiainen et al. 2011 [9] | Mouse (B6C3F1/ Crl) liver | MeDIP-chip | DNA methylation level in gene promoters | NA | Extracted from literarure |

**References**

1. Hughes ME, DiTacchio L, Hayes KR, Vollmers C, Pulivarthy S, et al. (2009) Harmonics of circadian gene transcription in mammals. PLoS Genet 5: e1000442.

2. Vollmers C, Gill S, DiTacchio L, Pulivarthy SR, Le HD, et al. (2009) Time of feeding and the intrinsic circadian clock drive rhythms in hepatic gene expression. Proc Natl Acad Sci U S A 106: 21453-21458.

3. Hughes M, Deharo L, Pulivarthy SR, Gu J, Hayes K, et al. (2007) High-resolution time course analysis of gene expression from pituitary. Cold Spring Harb Symp Quant Biol 72: 381-386.

4. Oster H, Damerow S, Hut RA, Eichele G (2006) Transcriptional profiling in the adrenal gland reveals circadian regulation of hormone biosynthesis genes and nucleosome assembly genes. J Biol Rhythms 21: 350-361.

5. Sharova LV, Sharov AA, Nedorezov T, Piao Y, Shaik N, et al. (2009) Database for mRNA half-life of 19 977 genes obtained by DNA microarray analysis of pluripotent and differentiating mouse embryonic stem cells. DNA Res 16: 45-58.

6. Huttlin EL, Jedrychowski MP, Elias JE, Goswami T, Rad R, et al. (2010) A tissue-specific atlas of mouse protein phosphorylation and expression. Cell 143: 1174-1189.

7. Lattin JE, Schroder K, Su AI, Walker JR, Zhang J, et al. (2008) Expression analysis of G Protein-Coupled Receptors in mouse macrophages. Immunome Res 4: 5.

8. Rey G, Cesbron F, Rougemont J, Reinke H, Brunner M, et al. (2011) Genome-Wide and Phase-Specific DNA-Binding Rhythms of BMAL1 Control Circadian Output Functions in Mouse Liver. PLoS Biol 9: e1000595.

9. Lempiainen H, Muller A, Brasa S, Teo SS, Roloff TC, et al. (2011) Phenobarbital mediates an epigenetic switch at the constitutive androstane receptor (CAR) target gene Cyp2b10 in the liver of B6C3F1 mice. PLoS One 6: e18216.
